# Neuromelanin-inspired metal-selenomelanin nanonetworks restore neuronal excitability in radiation-induced brain injury

**DOI:** 10.1126/sciadv.aei6265

**Published:** 2026-11-11

**Authors:** Ruotong Deng, Ke Li, Rui Duan, Xinyue Zhang, Botian Gu, Lanqun Mao, Wei Cao

**Affiliations:** Key Laboratory of Radiopharmaceuticals, Ministry of Education, College of Chemistry, Beijing Normal University, Beijing 100875, China.

## Abstract

Radiation-induced brain injury (RIBI) affects up to 30% of patients receiving cranial radiotherapy, representing an irreversible complication lacking effective therapies. Research advances are limited by poor blood-brain barrier drug penetration and overreliance on ex vivo pathology, which cannot resolve dynamic neuronal dysfunction. Inspired by neuromelanin, we fabricated metal-selenomelanin nanonetworks (MSMNs), combined with in vivo electrophysiology to investigate and mitigate radiation-triggered neural dysfunction. In 6-gray whole body–irradiated mice, MSMN normalized neuronal excitability and restored medium spiny neuron firing and striatal circuit activity, improving locomotor and anxiety-related behaviors. Besides central functional recovery, MSMN provides systemic radioprotection, ensuring full 30-day survival, protecting radiosensitive organs and facilitating hematopoietic recovery. This work identifies neuronal excitability as an underexplored therapeutic target for RIBI, establishing neuromelanin-mimetic selenomelanin nanonetworks as a bioinspired platform linking molecular radioprotection to circuit-level restoration for radiation-induced neurological injury.

## INTRODUCTION

Neural damage induced by ionizing radiation is often severe and largely irreversible (*1*, *2*), frequently leading to persistent cognitive decline, memory impairments, and neuropsychiatric dysfunction (*3*–*5*). Radiation is extensively used across medicine and industry (*6*–*13*), most notably as a cornerstone of clinical radiotherapy for head and neck and brain tumors (*6*–*9*). However, despite its therapeutic benefits, up to 30% of patients receiving cranial irradiation develop radiation-induced brain injury (RIBI) (*14*), a debilitating condition that profoundly compromises long-term quality of life (*3*–*5*). In contrast to other radiation-associated complications, such as dermatitis and enteritis, RIBI remains comparatively underexplored (*15*). This discrepancy arises not only from the anatomical and functional complexity of the brain, but also from fundamental barriers to both mechanistic investigation and therapeutic intervention. The blood-brain barrier (BBB) severely restricts drug delivery, while the highly heterogeneous neural microenvironment complicates precise mechanistic dissection. Moreover, radiation-induced neural dysfunction spans multiple scales—from oxidative damage to circuit-level alterations in neuronal excitability—requiring advanced and technically demanding methodologies for accurate characterization (*16*–*21*). These challenges have collectively hindered the development of effective therapeutic strategies.

To date, the only FDA-approved radioprotectant, amifostine, was primarily developed for protecting salivary glands rather than the central nervous system, and its clinical utility in RIBI remains limited. Although various approaches have been explored, including repurposing existing drugs (*22*–*26*) and developing new materials (*18*, *27*, *28*), current strategies are still far from satisfactory due to severe side effects, limited brain penetration, and transient efficacy (*22*, *29*). These limitations highlight an urgent need for fundamentally new design paradigms that can address both the biochemical and physiological dimensions of radiation-induced damage.

Melanin, a natural biopolymer with intrinsic radical-scavenging and metal-chelating capabilities (*30*, *31*), offers a compelling starting point. Inspired by melanized organisms that thrive in extreme radiation environments, such as tree frogs and fungi from Chernobyl (*32*, *33*). Melanin-based nanomaterials have emerged as a versatile and biocompatible platform for radioprotection (*34*–*37*). Recent advances in selenomelanin—an engineered analog incorporating selenium into melanin-like frameworks—have further amplified redox-regulatory capacity and radioprotective efficacy (*38*–*42*). Natural neuromelanin uses cysteinyl dopamine (DA) as its primary precursor (*43*), and metal ions participate in its biosynthetic process (*44*). Notably, neuromelanin has been closely linked to neuronal vulnerability and neurodegenerative processes (*40*, *45*, *46*), underscoring its intrinsic relevance to brain-specific injury. Inspired by native neuromelanin, we surmise that metal-integrated selenomelanin-based systems could provide an opportunity to interface with neuronal environments and mitigate RIBI at both molecular and functional levels.

Beyond therapeutic limitations, current RIBI research largely relies on ex vivo pathological assessments (*18*, *22*–*28*), such as histological staining, which lack temporal resolution, quantitative precision, and single-cell specificity. Mature neurons, being highly differentiated and nonproliferative, have long been traditionally considered relatively resistant to radiation. However, emerging evidence indicates that cranial irradiation can directly perturb neuronal firing patterns (*21*, *47*). This shift in understanding highlights neuronal excitability as a critical yet underexplored dimension of RIBI pathology and underscores the need for in vivo, neuron-level functional evaluation. Addressing this gap is essential for both elucidating disease mechanisms and developing next-generation neuroprotective strategies.

Here, we developed metal-selenomelanin nanonetworks (MSMNs) and used techniques such as in vivo multimodal electrophysiological recordings to investigate their protective effects against RIBI (Fig. 1). High-atomic metal bismuth provided enhanced radiation attenuation, while selenomelanin-based material catalytically neutralizes radiation-induced reactive oxygen species (ROS). Our materials demonstrate 100% survival and multiorgan protection in 6–gray (Gy) irradiation models. MSMN normalizes striatal neuronal circuit excitability and ameliorates radiation-induced locomotor and anxiety-related behavioral impairments, with regulatory effects on the firing activity of medium spiny neurons (MSNs) in the striatum. A hallmark finding of our study is that MSMN exerts robust neuroprotection against RIBI without crossing the BBB, challenging the conventional paradigm that therapeutic agents must penetrate the brain to treat brain injury. This reveals that non–brain-penetrating materials can still alleviate RIBI, paralleling recent breakthroughs in peripherally acting therapies for brain disorders (*48*–*50*). These results open avenues to non–brain-penetrating materials in the treatment of radiation-related neurological injuries and cross-organ regulatory mechanisms.

**Fig. 1.**
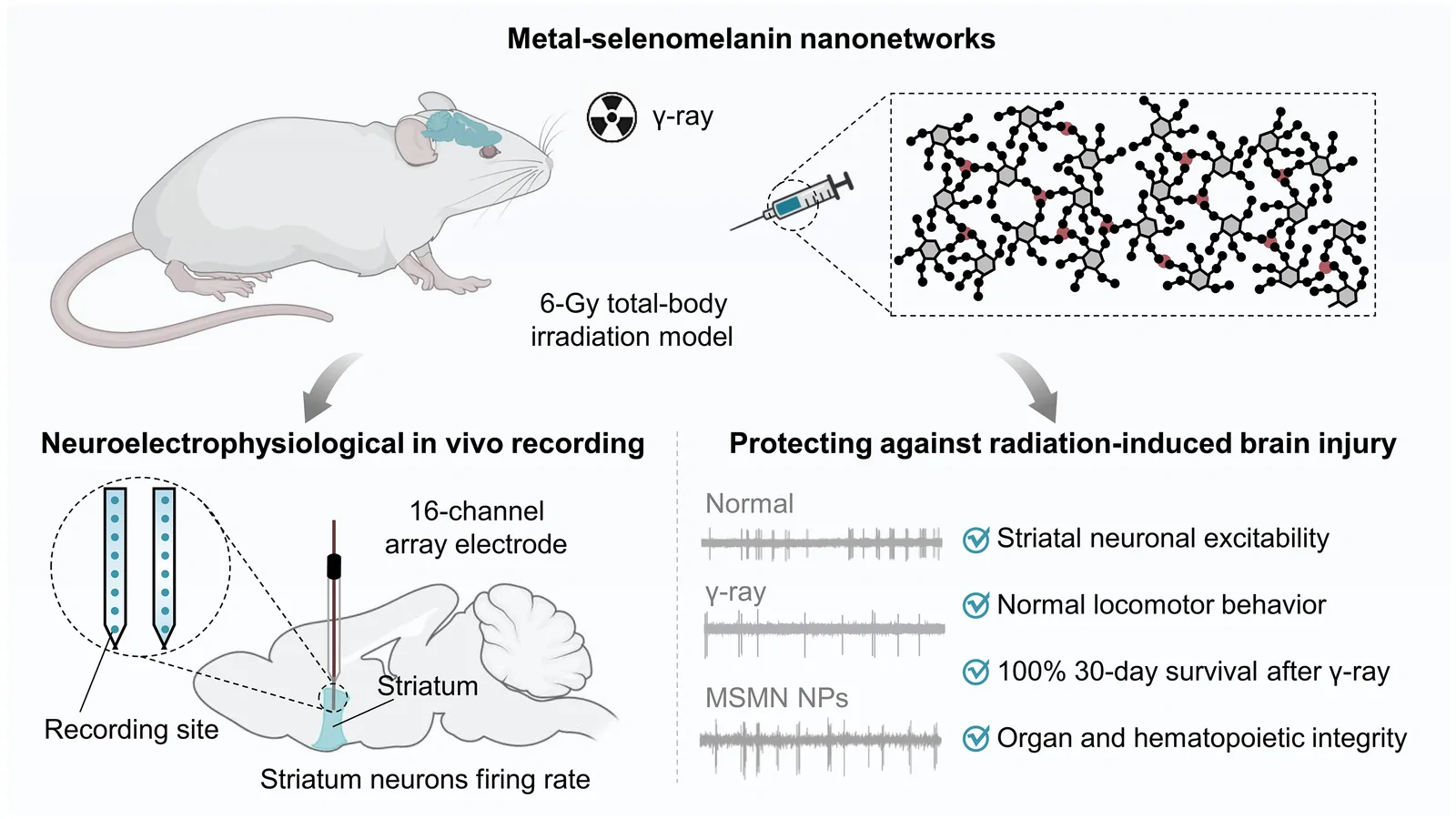
Schematic illustration of MSMNs restoring neuronal excitability in RIBI. Created in BioRender. Deng, R. (2026) https://BioRender.com/1dov0sh.

## RESULTS

### Construction of MSMNs

We used two synthetic routes to prepare the MSMN-1 and MSMN-2 (Fig. 2A). For MSMN-1, the oxidative polymerization of l-3,4-dihydroxyphenylalanine (l-DOPA) was first driven by permanganate in the presence of selenocystamine, followed by coordination of bismuth nitrate. MSMN-2 was synthesized by first conducting the base-catalyzed oxidation of DA hydrochloride with selenocystamine, and then introducing bismuth for coordination. Both processes yielded stable aqueous dispersions of dark brown nanoparticles, with metal-phenolic networks potentially exhibiting radiation shielding capacity (*37*, *39*, *51*, *52*) and ROS scavenging ability (*53*) (Fig. 2, B and C).

**Fig. 2.**
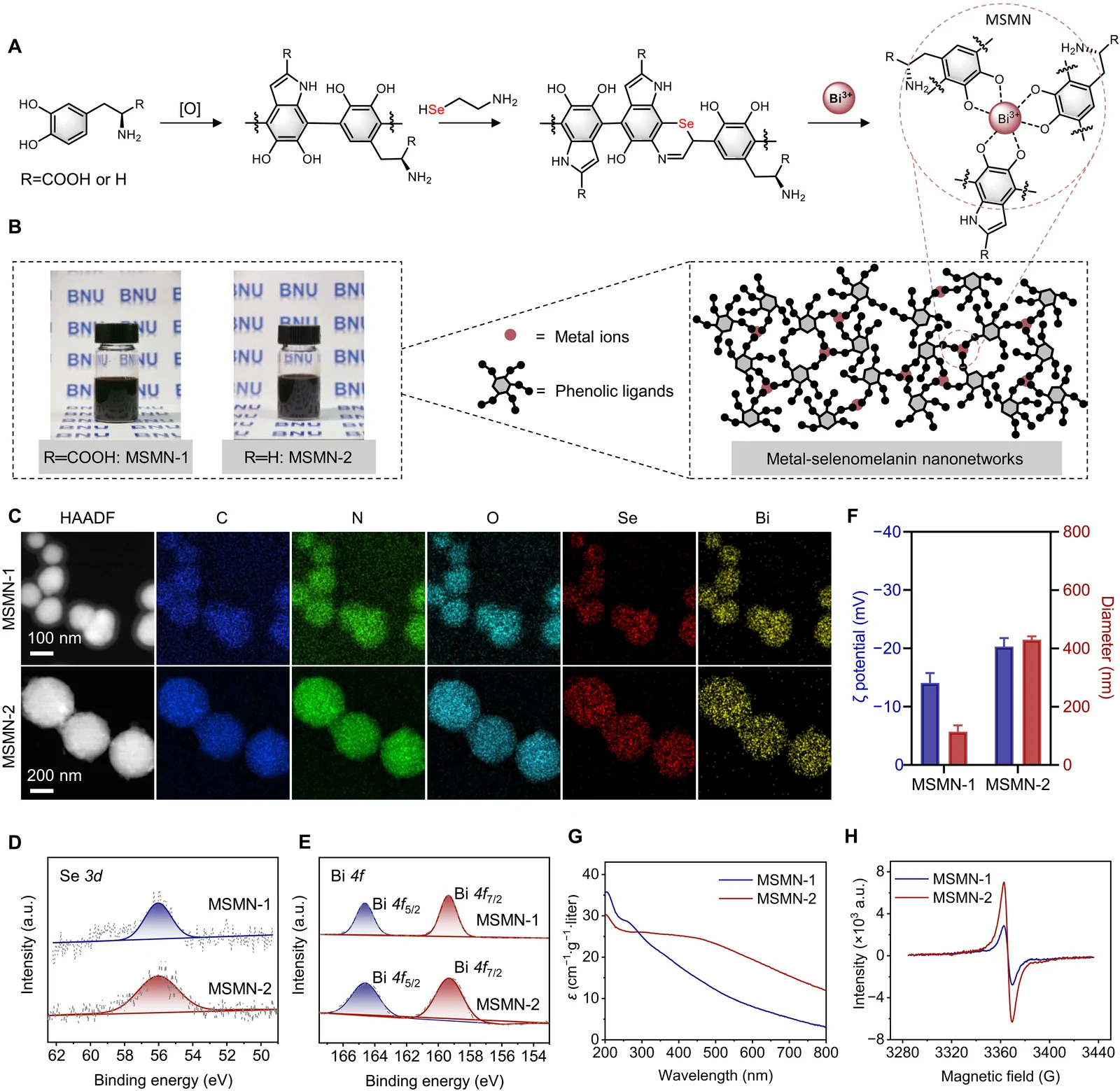
Synthesis and structural characterization of MSMN. (**A**) Chemical synthesis route of MSMNs. (**B**) Images of MSMN and their proposed structure of internal metal-phenolic networks. (**C**) High-angle annular dark-field (HAADF) and energy-dispersive x-ray spectroscopy (EDS) mapping images of MSMN-1 and MSMN-2. XPS spectra of Se *3d* (**D**) and Bi *4f* spectra (**E**) of MSMN-1 and MSMN-2. (**F**) Comparison of particle diameter (TEM results) and ζ potential (DLS results) of MSMNs. (**G**) UV-Vis spectra and (**H**) electron paramagnetic resonance spectra of MSMN-1 and MSMN-2. ε, mass absorption coefficient. a.u., arbitrary unit.

Energy-dispersive x-ray spectroscopy (EDS) elemental mapping confirmed the homogeneous distribution of C, N, O, Se, and Bi throughout the nanoparticles, indicating successful integration of metal into selenomelanin matrix (Fig. 2D). The Se *3d* x-ray photoelectron spectroscopy (XPS) spectra for both MSMN showed a single primary peak at a binding energy of ∼56.0 eV (Fig. 2D and figs. S1 and S2). The Bi *4f* spectra displayed doublet peaks at ∼159.3 eV (Bi *4f*_7_/_2_) and ∼164.6 eV (Bi *4f*_5_/_2_), characteristic of Bi(III), confirming stable integration of bismuth as Bi(III) (Fig. 2E). Transmission electron microscopy (TEM) revealed that MSMN exhibited a relatively uniform spherical morphology with average diameters of 114 ± 22 nm (MSMN-1) and 430 ± 12 nm (MSMN-2) (Fig. 2F). Dynamic light scattering (DLS) analysis corroborated these sizes in hydration state and further confirmed the negative ζ-potential (MSMN-1: −14.1 ± 1.7 mV, MSMN-2: −20.3 ± 1.5 mV) (Fig. 2F and figs. S3 and S4). Furthermore, MSMN showed a broad absorption spectrum spanning the ultraviolet to the near-infrared region and a persistent radical signal (Fig. 2, G and H), a hallmark of melanin-like materials (*40*).

### Bi doping powers radiation attenuation of MSMN

We next quantified the radiation shielding of MSMN via computed tomography (CT) (fig. S5). MSMN displayed markedly brighter signals (higher attenuation) than control melanins (Fig. 3A), which was further ascertained by quantitative analysis of x-ray attenuation coefficients (Fig. 3, B and C). To elucidate the mechanism underlying this enhancement, we calculated effective atomic number (*Z*_eff_) using the Glasser formula (*54*, *55*)

$$Z_{\text{eff}}={\left(\frac{\sum_{i}\omega_{i}Z_{i}^{4}}{\sum_{i}\omega_{i}Z_{i}}\right)}^{\frac{1}{3}}$$

where ω_i_ represents the mass percentage of element *i* and *Z_i_* is the atomic number (*Z*) of element *i*. MSMN series displayed substantially higher *Z*_eff_ values than SM (Fig. 3D and table S1). MSMN-1 exhibited a higher *Z*_eff_ than MSMN-2 due to its higher Bi loading (292.2 versus 106.7 mg·g^−1^; table S2) from more carboxyl groups. Monte Carlo simulations predict μ/ρ for the energy range of 10 to 100 keV, with cross sections tabulated as the contributions of photoelectric effect, Compton scattering, and Rayleigh scattering toward the total mass attenuation coefficient (Fig. 3, E to G). MSMN-1 and MSMN-2 exhibited higher μ/ρ values than SM-1 and SM-2 (Fig. 3E), consistent with their elevated *Z*_eff_. Deconvolution of the total attenuation (Fig. 3, F and G) revealed that the enhanced attenuation of MSMN-1 versus SM-1 is dominated by amplified photoelectric effect contribution, with small attenuation increases near the Bi K-edge (∼90.5 keV) and Se K-edge (∼12.6 keV). Since the photoelectric effect increases with *Z*_eff_ (μ/ρ ∝ *Z*_eff_*^n^*/*E*^3^, *n* ≈ 4 to 5) (*41*, *56*), Bi (*Z* = 83) in MSMN greatly strengthens energy absorption compared to Bi-free melanin. MSMN-2 and SM-2 yield identical component attenuation trends (fig. S6). As illustrated in Fig. 3H, Bi coordination mainly enhances photoelectric absorption and directly boosts the attenuation capacity of MSMN. In addition, Compton recoil electrons gradually lose energy while passing through the aromatic oligomers and π-electron systems of melanin, until trapped by stable free radicals in melanin (*37*, *41*, *57*, *58*). This controlled energy dissipation prevents secondary ionization and harmful reactive species generation, further reinforcing radiation shielding (*37*). Notably, the x-ray attenuation of MSMN can be optimized by increasing π-conjugation via reducing carboxyl side chains: At the same Bi mass concentrations (Fig. 3, I and J), MSMN-2 shows stronger attenuation than MSMN-1 due to its higher conjugation degree (*38*). Bismuth doping enhances MSMN’s radiation shielding capacity without impairing its outstanding antioxidant activity. Furthermore, the pronounced steric hindrance of carboxyl groups in MSMN-1 mitigates nanoparticle aggregation and facilitates interaction with 2,2-diphenyl-1-picrylhydrazyl (DPPH) radicals, resulting in a higher DPPH scavenging efficiency for MSMN-1 relative to MSMN-2 (fig. S7).

**Fig. 3.**
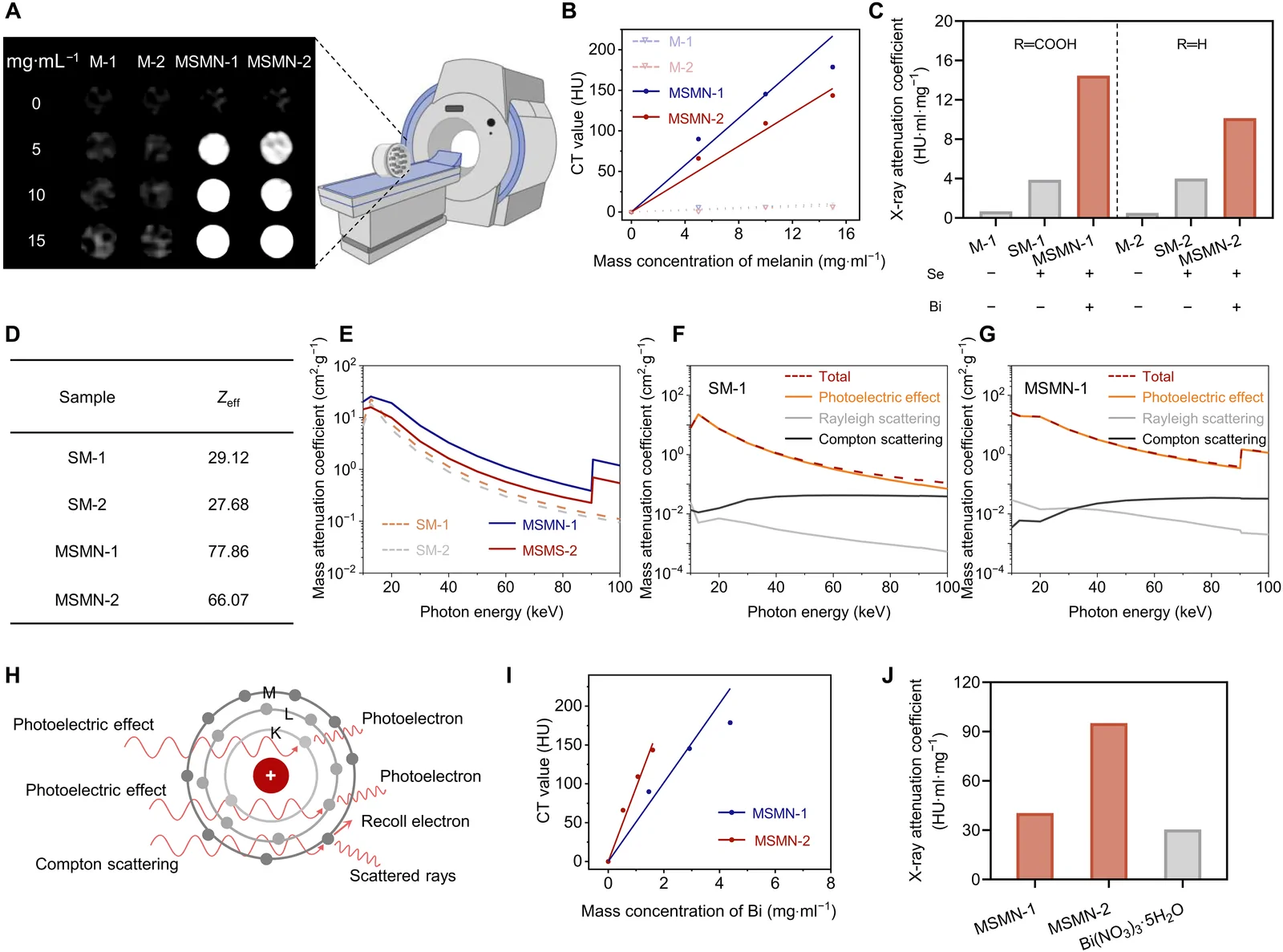
High-*Z* bismuth doping empowers MSMN with superior x-ray attenuation via enhanced photoelectric effect. (**A**) CT images of samples with different mass concentrations at 80 kVp. Created in BioRender. R. Deng (2026; https://BioRender.com/lcpoqmn). (**B**) CT values (HU: hounsfield units) of samples versus their mass concentrations at 80 kVp, with the corresponding linear fitting shown. Coefficient of determination (*R*^2^; the measure of fit of the linear regression model) of M-1, M-2, MSMN-1, and MSMN-2 are 0.856, 0.924, 0.994, and 0.984, respectively. (**C**) X-ray attenuation coefficients of samples [melanins M-1, M-2; selenomelanin (*38*) SM-1, SM-2], where the coefficient is derived from the slope of the linear fitting curve between the mass concentration of melanin nanoparticles (*x* axis) and the corresponding CT value (*y* axis). (**D**) Effective atomic number (*Z*_eff_) of samples. The *Z*_eff_ values were calculated on the basis of the mass percentage of each element using the Glasser formula. (**E**) Mass attenuation coefficients (μ/ρ) of SM-1, SM-2, MSMN-1, and MSMN-2 samples. (**F** and **G**) Component mass attenuation curves obtained by Geant4-based Monte Carlo simulation of (F) SM-1 and (G) MSMN-1. *y* axis are logarithmic scales. (**H**) Schematic illustration of the interaction mechanism between photons and MSMN. (**I**) CT values at 80 kVp versus the mass concentration of Bi in the samples, with the corresponding linear fitting shown. *R*^2^ of MSMN-1 and MSMN-2 are 0.971 and 0.984, respectively. (**J**) X-ray attenuation coefficients of bismuth-containing melanin samples, where the coefficient is calculated from the slope of the linear fitting curve between the mass concentration of Bi (*x* axis) and the corresponding CT value (*y* axis).

### Unlocking multifaceted cellular radioprotection mechanisms of MSMN

We further investigated the radioprotection of MSMN in HaCaT cells [a human adult keratinocyte cell line, a common radioprotection model (*38*, *41*, *59*, *60*)]. First, cytotoxicity assays confirmed the high biocompatibility of MSMN and a safe concentration of 5 μg·ml^−1^ was selected (fig. S8). Clonogenic survival assays revealed that MSMN-treated cells formed markedly more colonies than unprotected cells exposed to 10-Gy γ-ray (Fig. 4A). MSMN restored cells to normal proliferation levels across all tested cell densities (Fig. 4B).

**Fig. 4.**
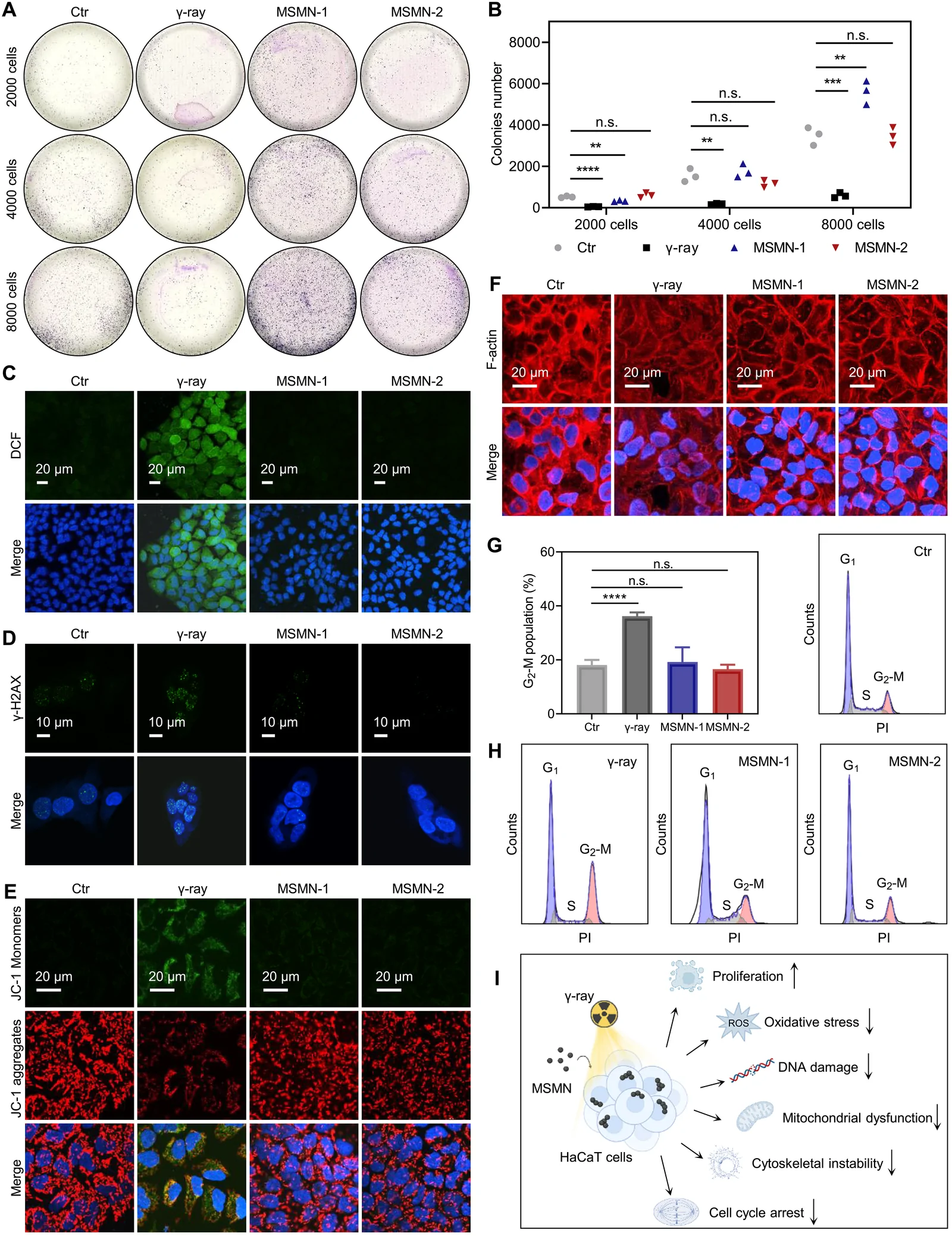
Multifaceted cellular radioprotective effect of MSMN. (**A**) Clonogenic assay after 10-Gy γ-ray in clonogenic cell survival assay, with an initial density of 2000/4000/8000 cells per well in six-well plates. Representative colony images were acquired using a Leica THUNDER DMi8. (**B**) Colonies numbers of (A). (**C**) CLSM images of live cells treated with 10-Gy γ-ray, showing DCF-DA ROS-positive signal (green). Cell nuclei were stained with Hoechst 33342 (blue). Scale bars, 20 μm. (**D**) DNA damage (γ-H2AX foci, green) of HaCaT cells treated with 10-Gy γ-ray. Cell nuclei were stained with DAPI (blue). Scale bars, 10 μm. (**E**) CLSM images of live cells treated with 10-Gy γ-ray, showing Actin-Tracker Red-594 F-actin–positive signal (red). Cell nuclei were stained with DAPI (blue). Scale bars, 20 μm. (**F**) CLSM images of mitochondrial membranes. JC-1 in high-potential healthy mitochondrial membranes fluoresces red at 561 nm excitation (corresponding to JC-1 aggregates), whereas JC-1 exists as monomers in low-potential mitochondrial membranes and fluoresce green at 488-nm excitation. Cell nuclei were stained with Hoechst 33342 (blue). Scale bars, 20 μm. (**G**) MSMN protected cells against cell cycle changes induced by γ-ray after 24 hours post-irradiation incubation. Error bars represent SD from three experiments (*n* = 3). n.s. means no statistical difference (*P* > 0.05) and ****P < 10^−4^, with reference to no material–treated and nonirradiated control (0 Gy), determined by Student’s two-tailed *t* test. (**H**) Cell cycle distribution plots at different γ-ray doses (0 and 10 Gy) and 24 hours post-irradiation incubation. Histograms are representative of three experiments. Note that (C) to (F) show selected fluorescence channel subsets. Full multichannel datasets are provided in figs. S9 to S12. (**I**) Schematic of radioprotection mechanisms of MSMN in HaCaT cells. Created in BioRender. Deng, R. (2026) https://BioRender.com/ldmfnfu.

Cellular exposure to high-energy radiation elicits excessive generation of toxic ROS, thereby triggering a cascade of severe cellular impairments and even culminating in cell death (*59*, *61*). 2′,7′-dichlorodihydrofluorescein diacetate (DCFH-DA) assay showed MSMN significantly reduced radiation-induced ROS levels, as reflected by decreased DCF fluorescence (Fig. 4C and fig. S9). γ-H2AX staining showed fewer foci in MSMN-treated cells compared with the unprotected group (Fig. 4D and fig. S10), suggesting that MSMN effectively mitigated radiation-induced DNA damage. JC-1 staining further revealed that MSMN treatment mitigated radiation-induced mitochondrial membrane potential change (Fig. 4E and fig. S11). MSMN also ameliorated the irradiation-induced deleterious cytoskeletal perturbations (Fig. 4F and fig. S12). The preservation of the actin cytoskeleton is critical for maintaining cellular homeostasis, morphology, polarity, and division (*62*). Meanwhile, MSMN effectively preserved normal cell cycle progression after 10-Gy γ-ray, with G_2_-M fractions of 18.1 ± 1.9%, 19.2 ± 5.5%, and 16.6 ± 1.6% in nonirradiated control, MSMN-1, and MSMN-2 groups, respectively, while unprotected irradiated cells exhibited prominent G_2_-M arrest at 36.2 ± 1.4% (Fig. 4, G and H). Collectively, MSMN elicited multifaceted cellular defense against radiation-induced damage, including oxidative stress, DNA damage, cytoskeletal instability, mitochondrial dysfunction, and cell cycle arrest, thus conferring enhanced cellular viability and proliferation (Fig. 4I).

### MSMN boosts long-term survival in TBI models

We next evaluated MSMN in murine (BALB/cAnNCrl, male, 6-week-old) models of total body irradiation (TBI) (Fig. 5A). In a 6-Gy model (Fig. 5, B and C, and fig. S13), the 30-day survival rate of unprotected mice (γ-ray group) was ∼12%, with a median survival time of 6 days. Strikingly, 100% 30-day survival was achieved with the administration of MSMN at a dose of 7.5 mg·kg^−1^ (Fig. 5, B and C). In addition, bismuth chelation significantly boosted the protection efficiency of selenomelanin (SM-1: ∼33.3% versus MSMN-1: 100% 30-day survival). Conventional radioprotector amifostine at a high dose (H-AMF: 100 mg·kg^−1^) exerted only a modest protective effect, with a 20% 30-day survival rate and median survival time of 6 days (Fig. 5, B and C), and log-rank test revealed no statistically significant difference in the survival curves between the H-AMF and γ-ray groups (Fig. 5B). These findings confirmed that MSMN surpass clinical radioprotector amifostine and overcome its lack of effective systemic radiation protection.

**Fig. 5.**
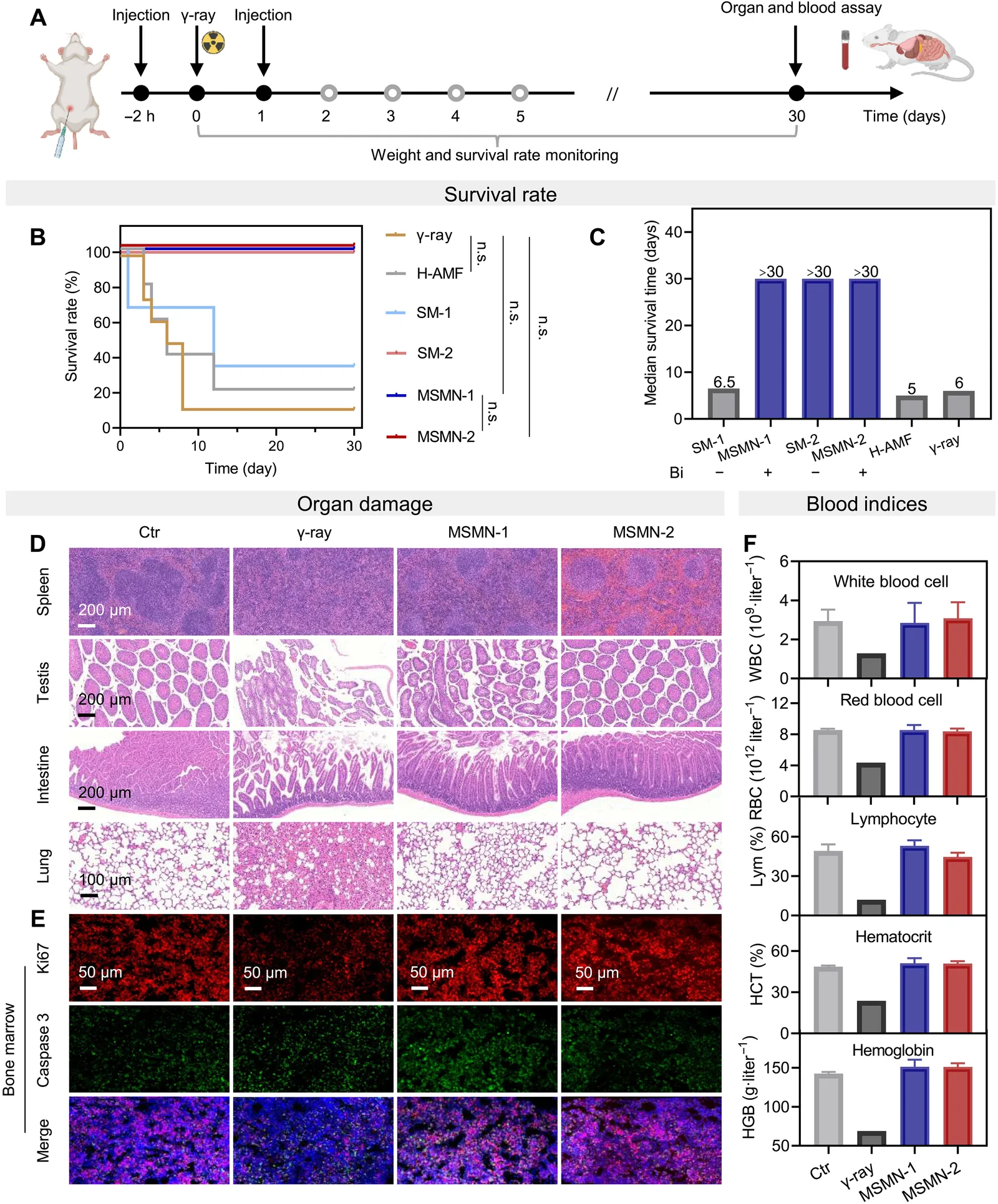
Boosted in vivo radioprotection of MSMN in TBI mice. (**A**) Schedule of in vivo radioprotection assays. Created in BioRender. Deng, R. (2026) https://BioRender.com/r566eev. (**B**) Monitoring of mice survival 30 days after irradiation in 6-Gy TBI. Statistical significance was determined by log-rank (Mantel-Cox) test. The mice were divided into six groups: (I) γ-ray (only 6-Gy TBI, *n* = 8), (II) H-AMF (high-concentration amifostine, 100 mg·kg^−1^, *n* = 5), (III) SM-1 (*n* = 3), (IV) SM-2 (*n* = 3), (V) MSMN-1 (*n* = 5), and (VI) MSMN-2 (*n* = 5). All irradiated groups [groups (I) to (VI)] received TBI (6 Gy, 2 Gy·min^−1^, 3 min). Group (II) received a single intraperitoneal dose of amifostine (100 mg·kg^−1^) 2 hours before the 6-Gy TBI. Groups (III) to (VI) were intraperitoneally injected with the respective test agent (3.75 mg·kg^−1^) 2 hours before and 24 hours after the 6-Gy TBI (cumulative dose of 7.5 mg·kg^−1^). (**C**) Median survival time (the time when half of the mice survived) of each group in 6-Gy TBI. “>30” denotes that survival exceeded 50% at the 30-day censoring point; hence, the median survival time was above 30 days. (**D**) Hematoxylin and eosin (H&E) staining of multiorgans 30 days after TBI. Scale bar of lung, 100 μm. Scale bars of other organs, 200 μm. (**E**) Immunofluorescence (IF) staining of marrow 30 days after TBI. Scale bars, 50 μm. Note that (E) shows selected fluorescence channel subsets. Full multichannel datasets are provided in fig. S16. The blank control panel (Ctr group) in (D) and (E) shown here were derived from the same set of control tissue sections also presented in Fig. 6. (**F**) Blood routine indices 30 days after TBI. WBC, white blood cell; RBC, red blood cell; Lym, lymphocyte; HCT, hematocrit; HGB, hemoglobin.

The histopathological analysis of radiosensitive organs at day 30 post–6-Gy TBI provided mechanistic insights into the survival benefit of MSMN (Fig. 5D and figs. S14 and S15). MSMN treatment well-preserved tissue architecture, whereas untreated irradiated mice showed severe injury: splenic atrophy with white pulp loss, testicular seminiferous tubule degeneration, intestinal villus blunting, and pulmonary pneumonitis (Fig. 5D). Decreased hematopoietic cell death marked by caspase 3 and enhanced proliferation indicated by Ki67 were observed in bone marrow specimens from MSMN-2–treated group compared with the irradiated mice (Fig. 5E and fig. S16). In sharp contrast, high-dose amifostine (H-AMF, 100 mg·kg^−1^) showed little protective effect on these organs and bone marrow (figs. S15 and S16). Peripheral blood counts in MSMN groups recovered to near-normal levels at 30 days (Fig. 5F), confirming effective hematopoietic reconstitution.

### MSMN features favorable metabolism and biocompatibility

The promising radioprotective efficacy of MSMN necessitate a thorough safety assessment, a critical step toward clinical translation. Single-photon emission CT (SPECT-CT) imaging showed a favorable metabolic profile of ^99m^Tc-labeled MSMN-2 (Fig. 6, A and B, and fig. S17). ^99m^Tc-labeled MSMN-2 was initially retained within the intraperitoneal cavity, manifested as prominent abdominal radioactivity signals at 0.5 hours post-intraperitoneal injection, with minimal apparent accumulation in vital organs, while a small fraction was translocated to the kidneys and bladder for early excretion. The nanoparticles were predominantly excreted via the renal-bladder pathway within 24 hours, suggesting a low risk of chronic accumulation in vital organs. Notably, free ^99m^TcO_4_^−^ conspicuously accumulated in thyroid and stomach at 0.5 to 8 hours and swiftly cleared to the bladder by 24 hours (*38*). In contrast, no significant thyroid distribution was observed in ^99m^Tc-MSMN-2, validating ^99m^Tc label remains stably associated with the melanin.

**Fig. 6.**
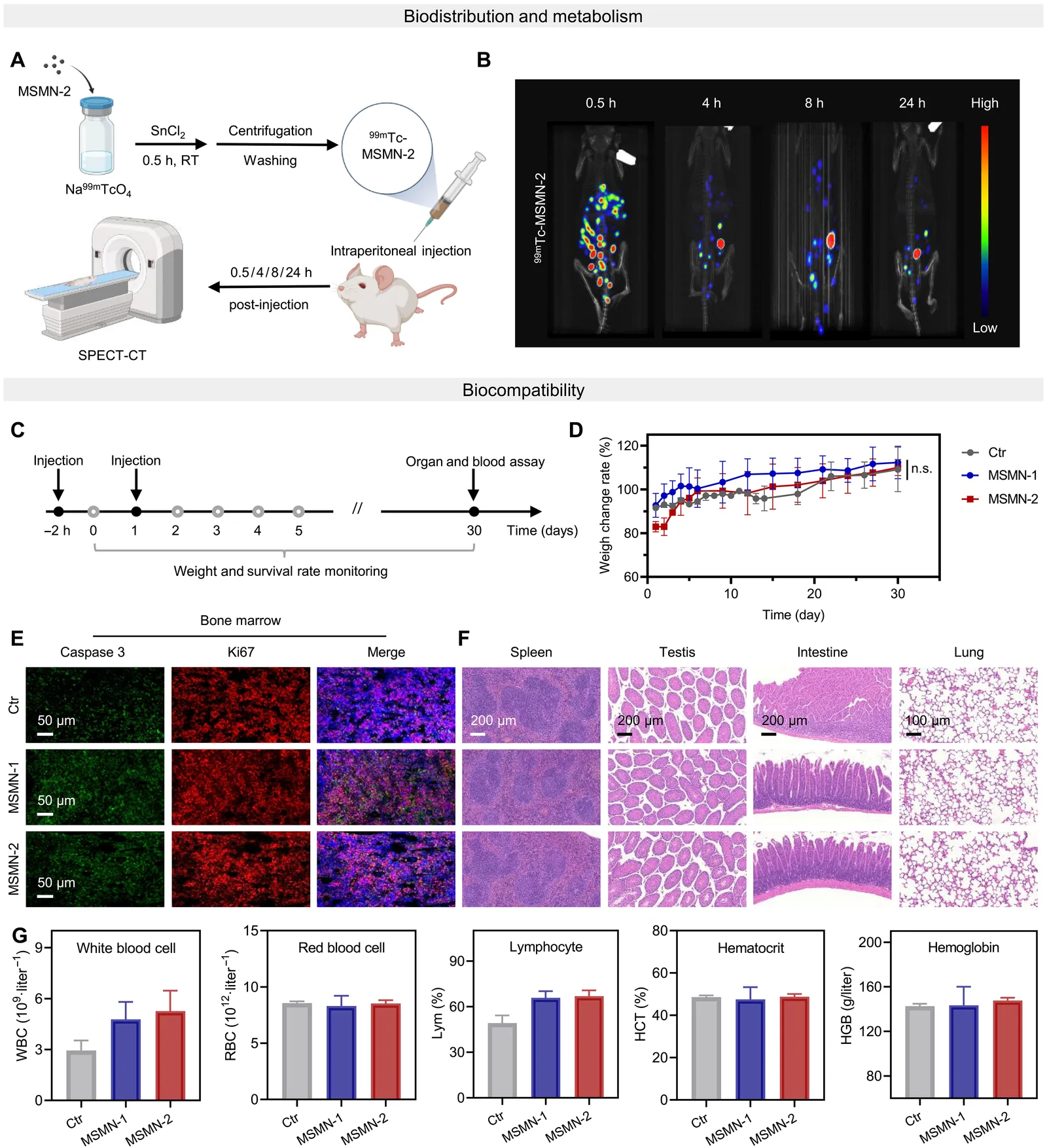
Favorable in vivo metabolism and biocompatibility of MSMN. (**A**) Schedule of in vivo metabolism imaging assays. Created in BioRender. Deng, R. (2026) https://BioRender.com/pump8h3. (**B**) SPECT-CT images of ^99m^Tc-MSMN-2 at various time points (0.5, 4, 8, and 24 hours) post–0.4 mCi ^99m^Tc-MSMN-2 administration; this imaging dose induces no extra radiation injury to mice (*74*). The high CT value white area in the image corresponds to the mouse ear tag and should not be mistaken for the thyroid. (**C**) Flowchart of in vivo biocompatibility assay. (**D**) Body weight change curves of mice during different treatments. (**E**) H&E staining of spleen, testis, lung and intestine at the 30-day censoring point in biosafety testing. Scale bar of lung, 100 μm. Scale bars of other organs, 200 μm. (**F**) Immunofluorescence staining of bone marrow at the 30-day censoring point in biosafety testing. Hematopoietic cell death was marked by caspase-3 and proliferation was indicated by Ki67. Scale bar, 50 μm. Note that (E) shows selected fluorescence channel subsets. Full multichannel datasets are provided in fig. S21. The blank control panel (Ctr group) in (E) and (F) shown here were derived from the same set of control tissue sections also presented in Fig. 5. (**G**) Blood routine indices at the 30-day censoring point in biosafety testing. The mice were divided into three group: (I) control (sham control, *n* = 3), (II) MSMN-1 [*n* = 3, received an intraperitoneal injection of MSMN-1 (7.5 mg·kg^−1^)], and (III) MSMN-2 [*n* = 3, received an intraperitoneal injection of MSMN-2 (7.5 mg·kg^−1^)]. h, hours.

We performed a 30-day biosafety study in healthy mice receiving the full therapeutic dose (7.5 mg·kg^−1^) of MSMN-1 or MSMN-2 (Fig. 6C). No mortality or significant body weight loss occurred in any treated group (Fig. 6D and fig. S18). Histological analysis of major organs (spleen, testis, lung, intestine, stomach, kidney, heart, and liver) at the end point revealed no pathological alterations compared to sham controls (Fig. 6E and figs. S19 and S20). Bone marrow cellularity and proliferation were normal, with no increase in apoptosis (Fig. 6F and fig. S21). Complete blood count analysis confirmed all hematological parameters remained within normal physiological ranges (Fig. 6G). These analyses demonstrated that MSMN had an excellent biocompatibility profile with no detectable acute or subchronic toxicity, meeting a key requirement for further clinical development as a safe radioprotective candidate.

### MSMN rescues striatal neuronal excitability to reverse radiation-elicited neurological dysfunction

We next investigated whether neuronal excitability underlies radiation-induced neurological dysfunction, and whether MSMN-mediated radioprotection translates into recovery of circuit-level activity and behavior. Using a 6-Gy TBI model, we performed combined behavioral and in vivo electrophysiological analyses focused on the striatum, a key hub for motor control (Fig. 7). In the open-field test, irradiated mice exhibited pronounced locomotor deficits and anxiety-like behavior, characterized by reduced exploration and avoidance of the center zone. In contrast, MSMN-2 group recovered to near-control levels by day 10, which persisted through day 30 (Fig. 7, B and C, and fig. S22), indicating effective mitigation of radiation-induced locomotor and behavioral impairments.

**Fig. 7.**
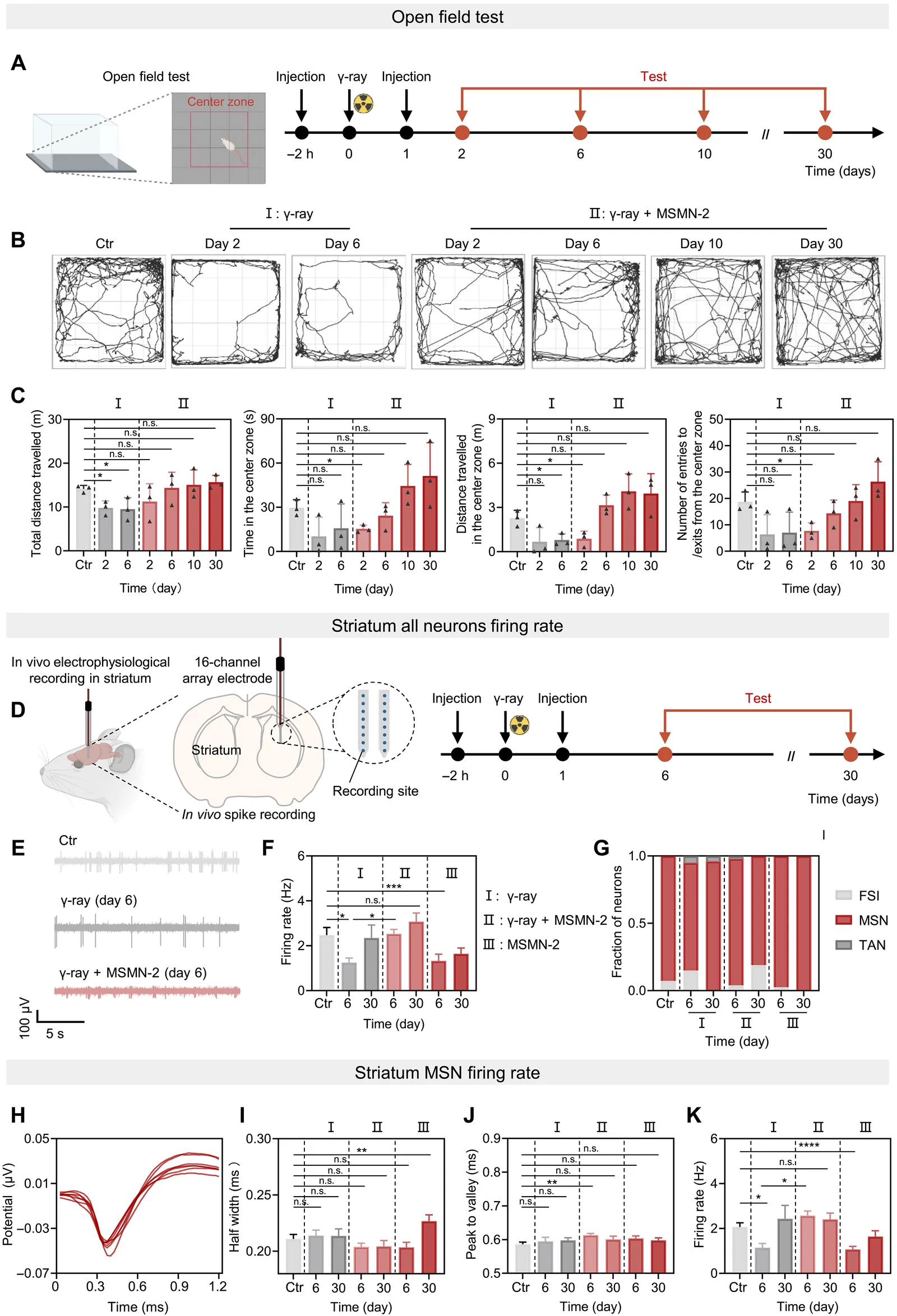
Behavioral and electrophysiological investigations revealed the neuroprotective effects and mechanisms of MSMN against RIBI. (**A**) Processes for open filed test. Created in BioRender. Deng, R. (2026) https://BioRender.com/fjkr1qy. (**B**) The activity tracks of the mice in the open field. (**C**) Total distance traveled, time in the center zone, distance traveled in the center zone, and number of entries to/exits from the center zone were analyzed. Grouping details of (B) and (C): *n* = 3. n.s., *P* > 0.05 and *P < 0.05, with reference to Ctr, determined by Student’s two-tailed *t* test. (**D**) Schematic illustration of electrophysiological recording. Created in BioRender. Deng, R. (2026) https://BioRender.com/fyvrkx5. (**E**) Representative action potential traces recorded in striatum neurons of mice with different treatments. (**F**) Comparison of firing rate recorded in striatum neurons of mice with different treatments. (**G**) Fraction of striatum neurons type of mice with different treatments. FSI, fast-spiking interneuron; MSN, medium spiny neuron; TAN, tonically active neuron. (**H**) Example waveforms of MSNs. Grouping details of (F-G): Ctr, *n* = 4; γ-ray (day 6), *n* = 3; γ-ray (day 30), *n* = 3; γ-ray + MSMN-2 (day 6), *n* = 3; γ-ray + MSMN-2 (day 30), *n* = 3; MSMN-2 (day 6), *n* = 3; MSMN-2 (day 6), *n* = 3. Comparison of waveform half-width (**I**) and peak to valley time (**J**) of MSN in the mice of different groups. n.s. *P* > 0.05 and **P < 10^−2^, with reference to Ctr, determined by Student’s two-tailed *t* test. (**K**) Comparison of firing rate recorded in striatum MSNs of mice with different treatments. Grouping details of (I) to (K): Ctr, *n* = 4; γ-ray (day 6), *n* = 3; γ-ray (day 30), *n* = 3; γ-ray + MSMN-2 (day 6), *n* = 3; γ-ray + MSMN-2 (day 30), *n* = 3; MSMN-2 (day 6), *n* = 3; and MSMN-2 (day 6), *n* = 3. n.s., *P* > 0.05; *P < 0.05; ***P < 10^−3^; and ****P < 10^−4^, determined by Student’s two-tailed *t* test. Error bars of (C), (F) and (G) and (I) to (K) represent standard error.

To establish the neuronal basis of this functional recovery, we performed in vivo multielectrode recordings in the striatum (Fig. 7D). Accumulating evidence indicates that the striatum constitutes an essential component of the motor control circuitry responsible for fundamental motor movements (*63*). Its dysfunction is a pathological hallmark of motor disorders, including Parkinson’s disease and Huntington’s disease (*64*). We hypothesized that MSMN-2 regulates striatum neuron activity to reverse the inhibitory effect of γ-ray on motor behavior. Irradiated striatal neurons showed marked hypoexcitability, while control neurons remained normal (Fig. 7, E and F). MSMN-2 treatment effectively restored striatal firing rates at day 6, rescuing the radiation-induced suppression of firing. Although partial recovery was observed in surviving irradiated mice by day 30 (Fig. 7F)—likely reflecting compensatory adaptation—the early restoration mediated by MSMN-2 highlights its direct neuroprotective effect.

Given that the striatum is predominantly composed of MSNs under physiological conditions (Fig. 7G) (*65*) and that pathological conditions are often associated with MSN degeneration and circuit remodeling, we next focused on MSN-specific activity. Spike sorting and single-unit clustering confirmed that neuronal subtype composition remained comparable across groups, enabling the direct analysis of MSN excitability. MSMN-2 treatment significantly restored MSNs firing rate reduced by γ-ray at day 6, without altering waveform characteristics, indicating preserved neuronal identity (Fig. 7, H to K). Quantitatively, γ-ray + MSMN-2 group (2.8 ± 0.2 Hz) exhibited a 2.8-fold higher firing rate than γ-ray group (1.0 ± 0.3 Hz), demonstrating robust functional rescue of MSMN-2 (*P* < 0.05) (Fig. 7K). γ-ray group (1.0 ± 0.3 Hz) showed a ∼ 50% reduction in firing rate compared with Ctr group (2.0 ± 0.2 Hz), reflecting severe radiation-induced neuronal hypoexcitability (*P* < 0.05). This restoration of MSN excitability is mechanistically and functionally significant, as reduced MSN activity is directly linked to impaired motor output (*66*).

Together, these results establish a causal chain in which γ-radiation reduces MSN excitability, impairing striatal circuit function and causing subsequent dysfunction, whereas MSMN-2 preserves MSN activity and alleviates these impairments. This study provides the first in vivo electrophysiological evidence for radiation-induced striatal neuronal hypoexcitability, a central node in motor control circuits. Prior electrophysiological investigations into radiation injury have primarily focused on neuronal firing abnormalities in the hippocampus and prefrontal cortex (*21*, *47*), emphasizing cognitive dysfunction, while overlooking striatal involvement. Our research fills the critical knowledge gap in RIBI research. The therapeutic intervention effectively rescues radiation-impaired striatal neuronal excitability and restores normal circuit function, distinguishing our work from previous studies that merely identified radiation-induced neuronal firing impairments without offering viable amelioration strategies. Dopaminergic neurons are believed to drive specific behaviors by rapidly modulating MSNs spiking activity in the striatum (*67*–*70*). However, it remains unclear whether the distinct firing rate patterns of MSNs, and their observed effects in radioprotective, are correlated with corresponding changes in DA levels. So, we conducted in vivo electrochemical monitoring of DA release with fast-scan cyclic voltammetry (FSCV). To evoke DA release (*71*), we injected 70 mM KCl (2 μl·min^−1^, 30 s) into the mice striatum, 300 μm away from the recording carbon fiber electrodes (CFEs) (fig. S23A). DA concentration was calculated using a calibration curve (fig. S23B). As shown in fig. S23 (C and D), unprotected brain striatum DA release was significantly reduced, whereas nonirradiated neurons maintained normal DA release. To investigated the protective effects of MSMN-2, we monitored DA release in striatum at various time points after irradiation (day 6, day 30), following their pretreatment with MSMN-2 (fig. S23, C and D). However, the protective effect of MSMN-2 at 6-day or 30-day postirradiation were less pronounced, indicating the MSN regulatory effect of MSMN-2 maybe through other chemical neurotransmission inputs, such as cortex.

## DISCUSSION

In summary, we report a neuromelanin-inspired metal-selenium–integrated selenomelanin nanonetwork as an effective strategy for mitigating RIBI. MSMN confers robust systemic radioprotection, achieving complete survival in a 6-Gy TBI model while preserving radiosensitive organs and hematopoietic function. Beyond conventional end points, MSMN restores striatal circuit function by normalizing neuronal excitability and rescuing the firing dynamics of MSNs, which translates into significant improvements in motor and anxiety-related behaviors.

Notably, despite their inability to penetrate the BBB, MSMN elicit pronounced neuroprotective effects, suggesting a noncanonical mode of action that does not rely on direct brain accumulation. This observation challenges the prevailing paradigm that effective interventions for central nervous system disorders must access the brain parenchyma and instead points to the existence of peripheral regulatory pathways capable of modulating neuronal function. In this context, emerging evidence of systemic-neural cross-talk, including gut-brain axis-mediated regulation, provides a plausible mechanistic framework (*50*, *72*, *73*). Together, this work establishes a noncanonical paradigm in which neuronal function can be restored through peripheral regulation, positioning nonbrain-penetrating nanomaterials as a viable strategy for treating radiation-induced and potentially broader neurological disorders.

## MATERIALS AND METHODS

### Ethical statement

Animal studies were performed in compliance with the guidelines of the Ethics Committee of Beijing Normal University and regulations on laboratory animals of Beijing Laboratory Animal Management Office (approval no. BNUCC-EAW-20240403-01). Six-week-old BALB/c (BALB/cAnNCrl) male mice were purchased from Charles River Laboratories (Beijing, China) [details: species: *Mus musculus* (mouse); strain: BALB/c (BALB/cAnNCrl); sex: male; age: 6 weeks old; genetic modification status: wild type, no genetic modification; RRID: RRID:IMSR_CRL:0001]. All mice were housed in a specific pathogen–free environment on a 12-hour light/12-hour dark cycle with the standard conditions: Temperature, 20° to 25°C; relative humidity, 40 to 70%. Mice were fed standard chow and distilled water throughout the experiments. All research was carried out according to relevant guidelines and regulations.

### Reagents and materials

l-DOPA was purchased from Leyan (Shanghai, China). Dopamine hydrochloride (DA·HCl) was purchased from Jiangsu Aikon. l-Selenocystine, tris(2-carboxyethyl) phosphine hydrochloride (TCEP·HCl), and bismuth nitrate pentahydrate (Bi(NO_3_)_3_·5H_2_O) were purchased from Aladdin. Selenocystamine dihydrochloride was purchased from Hwrk Chem. Potassium permanganate (KMnO_4_) was purchased from Beijing TongGuang Fine Chemicals Company. Ammonium hydroxide (NH_3_·H_2_O) was purchased from Macklin. Ethanol absolute (99.5%) was purchased from Innochem. CellTiter-Blue Reagent was purchased from Promega. DAPI (4′,6-diamidino-2-phenylindole) was purchased from Solarbio. Hoechst 33342 was purchased from Beijing LABLEAD Inc. Modified giemsa staining solution, cell cycle and apoptosis analysis kit, Actin-Tracker Red-594 kit, immunostaining fixative solution, Phospho-Histone H2A.X (Ser139) Rabbit Monoclonal Antibody (catalog no. AF1201), fluorescein isothiocyanate (FITC)–labeled goat anti-rabbit immunoglobulin G (IgG (H + L) (catalog no. A0562), ROS assay kit (DCF-DA, for cell), and mitochondrial membrane potential assay kit with JC-1 were purchased from Beyotime. All reagents and materials were used as received unless otherwise stated.

### Apparatus and characterization

TEM images were recorded using a FEI Tecnai Spirit 120 kV TEM at Tsinghua University. The zeta potential and DLS size of the nanoparticles in water were obtained on a Zetasizer Lab (Malvern Panalytical). EDS mapping images were captured with a FEI Talos F200S. UV-Visible (UV-Vis) absorbance measurement was carried out on a Cary 60 UV-Vis Spectrophotometer (Agilent Technologies). XPS spectra were analyzed by a Thermo Scientific K-Alpha. Inductively coupled plasma optical emission spectrometry (ICP-OES) was performed on an Agilent 5110 ICP-OES and a Thermo iCAP 7400 ICP-OES at Tsinghua University. Electron paramagnetic resonance spectra were measured on a Bruker EMXplus-6/1. CT images and Hounsfield Unit values were detected by a Philips IQon Spectral CT in Beijing Tongren Hospital.

Confocal laser scanning microscopy (CLSM) images were acquired using a Nikon A1R microscopy system at Beijing Normal University and a Multi-SIM AXR multimodal super-resolution confocal microscope at Tsinghua University. Flow cytometry data were recorded via a BD Accuri C6 Plus Flow cytometer. The fluorescence intensity of 96-well plates was monitored with the microplate reader Tecan Infinite M200 PRO. Cells were irradiated by ^60^Co gamma irradiator (GM-11-03-A, Beijing Gamma high-tech Co. Ltd) in Beijing Normal University. Na^99m^TcO_4_ was obtained from a ^99^Mo/^99m^Tc generator provided by Beijing Senke Pharmaceutical Co. Ltd. TriFoil Triumph II microSPECT/CT equipment (Trifoil) was used for imaging studies. Radioactivity was determined on a PerkinElmer system (WIZARD^2^ 2480 Automatic γ-Counter).

### Experimental methods

#### Preparations of melanin nanoparticles

MSMN-1: First, l-DOPA solution (0.06 mmol) was mixed with 0.2 M KMnO_4_ to form eumelanin seeds. Next, selenocysteamine solution (0.06 mmol) was added to the reaction flask. After overnight reaction, the product was collected by centrifugation and purified by washing with HCl solution. One equivalent of Bi(NO_3_)_3_·5H_2_O was added to the aforementioned centrifuged product and stirred for 3 hours. The product was collected by centrifugation and washed with ultrapure water three times.

MSMN-2: 99.5% ethanol absolute, ultrapure water, and 28 to 30% NH_3_·H_2_O solution were added to a flask and stirred vigorously. Dopamine hydrochloride solution (0.105 mmol) was added to the mixture. Next, selenocysteamine solution (0.105 mmol) was added to the reaction flask. After overnight reaction, the product was collected by centrifugation and washed with ultrapure water for three times. One equivalent of Bi(NO_3_)_3_·5H_2_O was added to the aforementioned centrifuged product and stirred for 3 hours. The product was collected by centrifugation and washed with ultrapure water three times.

#### X-ray attenuation capability

Philips IQon Spectral CT in Beijing Tongren Hospital was used to acquire the CT images and Hounsfield Unit values. The CT images were further analyzed using PmsDView software. Parameters of imaging are 80 kVp and 10 mA. To evaluate the CT signals in vitro under different concentrations of melanin and monomer, melanin suspension and monomer solution in ultrapure water were filled into 1.5-ml centrifuge tubes for CT tests.

#### Evaluations of mass attenuation coefficients

Using the open-source software Geant4 toolkit simulation code based on the Monte Carlo algorithm to simulate the three samples, we simulated monoenergetic rays from 10 to 100 keV, detected the number of photons that passed through the three samples and reached the detection region, and then evaluated the mass attenuation coefficient of each sample according to the Beer-Lambert law by using the initial and final photon intensities. *y* axes are logarithmic scales.

#### DPPH scavenging assay

A 100 μl of melanin suspension (0.04, 0.20, and 0.50 mg·ml^−1^) in ultrapure water (or 100 μl ultrapure water) was mixed with 1.8 ml of a 0.2 mM DPPH solution in 95% ethanol. The scavenging activity was evaluated by monitoring the absorbance decrease at 516 nm over 120 min. DPPH radical scavenging activity was calculated as

$$I=\left[1-\left(A_{i}-A_{j}\right)/A_{c}\right]*100\%$$ (1)

In Eq. (1), *A*_c_ is the absorbance of DPPH solution without melanin, *A*_i_ is the absorbance of melanin mixed with the DPPH solution, and *A*_j_ is the absorbance of melanin without DPPH.

It should be noted that MSMN-1 and MSMN-2 were the experimental materials adopted in this study. Data of M-1 and M-2 were only cited for comparative analysis (*38*). All datasets were obtained under consistent experimental conditions.

### Cell experiment

#### Cell culture

Human (*Homo sapiens*) spontaneously immortalized keratinocyte cell line HaCaT (supplier: Wuhan Sunncell Biotechnology Co. Ltd.; catalog number: SNL-163; universal RRID for HaCaT cell line: RRID: CVCL_0038). Cells were cultured in Dulbecco’s modified Eagle’s medium (VivaCell) containing 10% (v/v) fetal bovine serum and 1% antibiotics (penicillin-streptomycin) with high glucose and maintained at 37°C in 5% CO_2_ with a relative humidity of 95%.

#### Cellular biocompatibility

Briefly, HaCaT cells were seeded in 96-well plates at a density of 10,000 cells per well for 24 hours. The cells were then treated with melanin at different concentrations for another 24 hours. After the incubation period, the cells were washed three times with phosphate-buffered saline (PBS), and then CellTiter-Blue at 10% (v/v) in complete media was added to each well and incubated for 2 hours at 37°C to allow the live cells to convert resazurin to fluorescent resorufin. The fluorescent signal was then analyzed with excitation wavelength at 560 nm and emission wavelength at 600 nm by a plate reader. Untreated cells in complete medium were used as a blank control. Viability is reported as a percentage of untreated cells, averaged five biological repeats (*n* = 5).

#### Colony formation assay

HaCaT cells were planted in 6-well plates with 2000, 4000, and 8000 cells per well. Then, melanin (4 μg·ml^−1^) was added and incubated for 24 hours. Then, 10-Gy γ-ray radiation was applied. All irradiation was delivered at 10 Gy·min^−1^. On the seventh day after irradiation, the cells were washed with PBS and fixed with methanol for 10 min, and Giemsa stain (1:9) was applied for 30 min.

#### ROS scavenging experiments on HaCaT cells

HaCaT cells were plated at 50,000 cells per well in glass bottom dishes and seeded for 24 hours before treatment with melanin (4 μg·ml^−1^) for another 24 hours. Then, the cells were treated with 10-Gy γ-ray irradiation. All irradiation was delivered at 10 Gy·min^−1^. After incubation for 24 hours, the cells were washed two times with PBS and then treated with 10 μM DCF-DA and incubated at 37°C for 20 min. Next, the loading buffer was removed, and cells were returned to the growth medium. A drop of Hoechst 33342 dye was added to 0.5-ml media for the nucleus staining before imaging by CLSM. The following parameters are ROS probe scan excitation wavelength of 488 nm, detection wavelength of 510 to 550 nm, Hoechst 33342 scan excitation wavelength of 405 nm, and detection wavelength of 420 to 480 nm.

#### γ*-H2AX assay*

HaCaT cells were planted at 50,000 cells per well in glass bottom dishes and seeded for 24 hours before treatment with melanin (4 μg·ml^−1^) for another 24 hours. Next, the loading buffer was removed, and cells were returned to the growth medium. Then, the cells were treated with 10-Gy γ-ray irradiation. All irradiation was delivered at 10 Gy·min^−1^. After incubation for 24 hours, the cells were fixed with 4% paraformaldehyde for 10 min, followed by 0.1% Triton X-100 for 10 min, and then blocking solution for immunofluorescence staining for 20 min. Next, Phospho-Histone H2A.X (Ser^139^) Rabbit Monoclonal Antibody (1:1000) was incubated at room temperature for 3 hours, followed by FITC-labeled goat anti-rabbit IgG (H + L) (1:500) at 37°C for 1 hour. Last, DAPI was added and incubated for 10 min. The following parameters are γ-H2AX scan excitation wavelength of 488 nm, detection wavelength of 510 to 550 nm, DAPI scan excitation wavelength of 405 nm, and detection wavelength of 420 to 480 nm.

#### F-actin assay

HaCaT cells were planted at 50,000 cells per well in glass bottom dishes and seeded for 24 hours before treatment with melanin (4 μg·ml^−1^) for another 24 hours. Next, the loading buffer was removed, and cells were returned to the growth medium. Then, the cells were treated with 10-Gy γ-ray irradiation. All irradiation was delivered at 10 Gy·min^−1^. After incubation for 24 hours, the cells were fixed with immunostaining fixative solution for 10 min and washed three times with 0.1% Triton X-100 for 5 min each. Next, Actin-Tracker Red-594 was incubated at room temperature for 60 min. Last, DAPI was added and incubated for 10 min. The following parameters are F-actin probe scan excitation wavelength of 561 nm, detection wavelength of 570 to 625 nm, DAPI scan excitation wavelength of 405 nm, and detection wavelength: 420 to 480 nm.

#### JC-1 assay

HaCaT cells were planted at 50,000 cells per well in glass bottom dishes and seeded for 24 hours before treatment with melanin (4 μg·ml^−1^) for another 24 hours. Next, the loading buffer was removed, and cells were returned to the growth medium. Then, the cells were treated with 10-Gy γ-ray irradiation. All irradiation was delivered at 10 Gy·min^−1^. After incubation for 24 hours, the cells were washed once with PBS and added 0.5 mL of culture medium (which can contain serum and phenol red). A 0.5 ml of JC-1 staining solution [diluted by adding 5 μl of JC-1 (200×) to 1 ml of JC-1 buffer] was added to the cells. The cells were then incubated at 37°C for 20 min in a cell incubator and washed twice with JC-1 buffer. Last, 1 ml of culture medium (which can contain serum and phenol red) was added. A drop of Hoechst 33342 dye was added to 0.5-ml media for the nucleus staining before imaging by CLSM. The following parameters are JC-1 aggregates scan excitation wavelength of 561 nm and detection wavelength of 570 to 625 nm, JC-1 monomers scan excitation wavelength of 488 nm and detection wavelength of 510 to 550 nm, and Hoechst 33342 scan excitation wavelength of 405 nm and detection wavelength: 420–480 nm.

#### Cell cycle studies

HaCaT cells were plated at a density of 50,000 cells per well in 12-well plates and cultured for 24 hours. After incubating for 24 hours, melanin (4 μg·ml^−1^) was added to each well for 24 hours. Then, 10-Gy γ-ray radiation was applied (10 Gy·min^−1^). The cells were incubated for another 24 hours. The cells were then harvested, fixed, and stained according to a technical manual of cell cycle and apoptosis analysis kit for flow cytometry.

### Animal assay

All animal experiments were conducted in compliance with protocols approved by the Institutional Animal Care and Use Committee of Beijing Normal University. BALB/c (BALB/cAnNCrl) male mice (6 weeks old, ∼19.0 g) were purchased from Beijing Vital River Laboratory Animal Technology Co. Ltd. and were acclimatized for 7 days in a controlled environment (22° ± 1°C, 45 ± 5% humidity, 12-hour light/12-hour dark cycle). Sample size determination: Sample sizes were selected on the basis of common standards for mouse whole-body irradiation survival and neurobehavioral and electrophysiological assays in radioprotection research. Randomization: Random group allocation was performed, and mice were randomly assigned to different treatment groups after matching body weight. Inclusion/exclusion criteria: Preset inclusion criteria are as follows: healthy 6-week-old male BALB/c mice with normal body weight and no spontaneous disease; exclusion criteria: mice with weight loss >10% before irradiation. All in vivo animal procedures were conducted strictly following the ARRIVE 2.0 guidelines.

#### 6-Gy TBI model

The mice were divided into six groups: (I) γ-ray (only 6-Gy TBI, *n* = 8), (II) H-AMF (high-concentration amifostine, 100 mg·kg^−1^, *n* = 5), (III) SM-1 (*n* = 3), (IV) SM-2 (*n* = 3), (V) MSMN-1 (*n* = 5), and (VI) MSMN-2(*n* = 5). All irradiated groups [groups (I) to (VI)] received TBI (6 Gy, 2 Gy·min^−1^, 3 min). Group (II) received a single intraperitoneal dose of 100 mg·kg^−1^ amifostine 2 hours before the 6-Gy TBI. Groups (III) and (VI) were intraperitoneally injected with 3.75 mg·kg^−1^ of the respective test agent 2 hours before and 24 hours after the 6 Gy total body irradiation (cumulative dose 7.5 mg·kg^−1^). It should be noted that H-AMF, SM-1, MSMN-1, and MSMN-2 in the 6-Gy model were the experimental materials adopted in this study. Data of other groups were only cited for comparative analysis (*38*). All datasets were obtained under consistent experimental conditions. Daily monitoring was conducted, and weight as well as survival within 30 days after total body irradiation were recorded. Blood, organs, and bone marrow collected at day 30 postirradiation were harvested for further analysis.

#### SPECT-CT imaging assays

A 0.1 ml of SnCl_2_ solution [5 mg·ml^−1^; prepared with HCl solution (0.1 mol·liter^−1^)] and 1 mCi of Na^99m^TcO_4_ were added to 1.5 ml of melanin (0.75 mg·ml^−1^). The mixture was stirred at room temperature for 30 min, then ultrafiltrated, and centrifuged for three times (5 min each time) to remove unbound nuclides. The final product ^99m^Tc-melanin was obtained and its radioactivity was determined. BALB/c mice were intraperitoneally injected with 0.4 mCi fresh Na^99m^TcO_4_ solution (free ^99m^TcO_4_^−^) or ^99m^Tc-Melanin and imaged by micro SPECT-CT at 0.5, 2, 8, and 24 hours.

#### Biosafety evaluation

The mice were divided into three groups: (I) control (sham control, *n* = 3), (II) MSMN-1 (*n* = 3), and (III) MSMN-2 (*n* = 3). Groups (II) and (III) each received an intraperitoneal injection of 7.5 mg·kg^−1^ of the respective test agent. Daily monitoring was conducted, and weight as well as survival within 30 days after injection were recorded. Blood, organs, and bone marrow collected at day 30 postinjection were harvested for further analysis. The remaining materials used in the animal experiments have been verified for their biological safety (*38*, *60*).

#### Open-field test

Mice were placed in an experimental box (40 cm by 40 cm by 40 cm), and their behaviors were recorded for 10 min. The total distance traveled, speed, and time spent in the central area were analyzed using ANY-maze Video Tracking System (7.33 version). Before all behavioral experiments, mice were allowed to adapt to the experimental room for 30 min to 1 hour. After each mouse was tested, the equipment was cleaned with 75% ethanol. The experimental process was kept quiet and with uniform lighting. The mice were divided into three groups: (I) Ctr: sham control, *n* = 3; (II) γ-ray: mice with γ irradiation only, *n* = 3; and (III) γ-ray + MSMN-2: mice with MSMN-2 treatment only, *n* = 3. All irradiated groups [groups (II) and (III)] received TBI (6 Gy, 2 Gy·min^−1^, 3 min). Group (III) was intraperitoneally injected with MSMN-2 (3.75 mg·kg^−1^) 2 hours before and 24 hours after the 6-Gy TBI (cumulative dose 7.5 mg·kg^−1^). In group (II), the open-field test was conducted on the second and sixth days after irradiation. In group (III), the open-field test was conducted on the 2nd, 6th, 10th, and 30th days after irradiation.

#### Electrophysiological recording in striatum of anesthetized mice

For electrophysiology recording, mice were anesthetized with urethane (1 g·kg^−1^ body weight, i.p.) and positioned on a stereotactic frame. After the skull was exposed, a hole was made above the right striatum, a 16-channel tungsten array electrode (diameter: 50 μm, probe spacing: 200 μm, array: 2 × 8, impedance: 500 to 800 kΩ) was slowly implanted into the striatum [anteroposterior (AP): 0 to 1 mm, mediolateral (ML): 1.5 to 2.5 mm, dorsoventral (DV): 2.2 to 3.5 mm]. Small stainless screws were used as reference and ground electrodes, which were fixed in the skull above the cerebellum. Electrodes were connected to 16-channel headstage with a preamplifier and an Omniplex neural data acquisition system (Plexon). The neural activity was digitized at 40 kHz and high-pass filtered from 300 Hz for spike analysis. The mice were divided into seven groups: (I) Ctr: sham control, *n* = 4; (II) γ-ray (day 2): mice with γ irradiation only, tested at 2 days postirradiation, *n* = 3; (III) γ-ray (day 6): mice with γ irradiation only, tested at 6 days post irradiation, *n* = 3; (IV) γ-ray + MSMN-2 (day 6): mice with γ irradiation + MSMN-2 treatment, tested at 6 days postirradiation, *n* = 3; (V) γ-ray + MSMN-2 (day 30): mice with γ irradiation + MSMN-2 treatment, tested at 30 days postirradiation, *n* = 3; (VI) MSMN-2 (day 6): mice with MSMN-2 treatment only, tested at 6 days postinjection, *n* = 3; and (VII) MSMN-2 (day 30): mice with MSMN-2 treatment only, tested at 30 days postinjection, *n* = 3. All irradiated groups [groups (II) to (V)] received total body irradiation (6 Gy, 2 Gy·min^−1^, 3 min). Groups (IV) to (VII) were intraperitoneally injected with MSMN-2 (3.75 mg·kg^−1^) 2 hours before and 24 hours after the 6-Gy TBI (cumulative dose of 7.5 mg·kg^−1^).

#### Analysis of neuronal activity

Spike analysis was conducted using Off-Line Sorter version 3 software (Plexon, Dallas, TX, USA). Spikes with a signal-to-noise ratio exceeding three standard deviations were selected and sorted based on the waveform and the first three principal components of the spike waveform on each channel. Waveforms with similar principal components were clustered using the *K*-means sorting method. The isolated cluster was regarded as a single unit recorded from the same neuron. Spikes with interspike intervals <2 ms were discarded. Firing rate histograms were generated using NeuroExplorer software (Plexon, Dallas, TX, USA, version 5.0).

### Fabrication of CFEs for FSCV

The carbon fibers with the diameter of 7 μm were cut under a microscope to expose 150 μm in length. Before use, the electrodes were electrochemically activated by applying +1.5 V (versus Ag/AgCl) for 10 s in 1 M NaOH with potential-controlled amperometry and then using high-speed cyclic voltammetry in Artificial Cerebrospinal Fluid (aCSF) solution until a stable voltammogram was obtained.

### In vivo FSCV monitoring of dopamine (DA) release

Briefly, mice were anesthetized with urethane (1 g·kg^−1^ body weight, intraperitoneal injection) and positioned on a stereotactic frame. A CFE was implanted into striatum (AP: 0 to 1 mm, ML: 1.5 to 2.5 mm, and DV: 2.2 to 3.5 mm). DA release was induced with KCl solution (70 mM). For local injection, a tube was implanted into the same depth of the CFE with a distance of 300 μm from the CFE. During the surgery, the body temperature of the mice was maintained at 37°C with a heating pad. DA concentration changes were monitored by recording the oxidation current at the peak potential across successive voltammetric scans, enabling real-time quantification of DA release dynamics.

### Statistical analysis

All data are presented as their means with SD. Error bars indicate ± SD. Statistical significance was determined by log-rank (Mantel-Cox) test or two-tailed Student’s *t* test. n.s. means no statistical difference (*P* > 0.05), *P < 0.05, **P < 10^−2^, ***P < 10^−3^, ****P < 10^−4^, and *****P < 10^−5^. Pre-established inclusion and exclusion criteria were defined for in vivo mouse experiments. No cell samples, animal subjects, or data points were omitted from statistical analysis; no attrition or intentional data exclusion occurred across all experiments. Unless otherwise specified, all data presented in this manuscript are derived from biological replicates.
