# Supplementary material for: Neuromelanin-inspired metal-selenomelanin nanonetworks restore neuronal excitability in radiation-induced brain injury
Source: Sci Adv. 2026 Nov 11;12(46):eaei6265. doi: 10.1126/sciadv.aei6265 (PMC13754026; doi:10.1126/sciadv.aei6265)
Supplement: Supplementary file 1 [file sciadv.aei6265_sm.pdf]

Science Advances
AAAS

Supplementary Materials for

Neuromelanin-inspired metal-selenomelanin nanonetworks restore neuronal excitability in radiation-induced brain injury

Ruotong Deng *et al.*

Corresponding author: Lanqun Mao, lqmao@bnu.edu.cn; Wei Cao, caowei@bnu.edu.cn

Sci. Adv. **12**, eaei6265 (2026)
DOI: 10.1126/sciadv.aei6265

This PDF file includes:

Figs. S1 to S23
Tables S1 to S3

Supplementary Information

Supplementary Figures

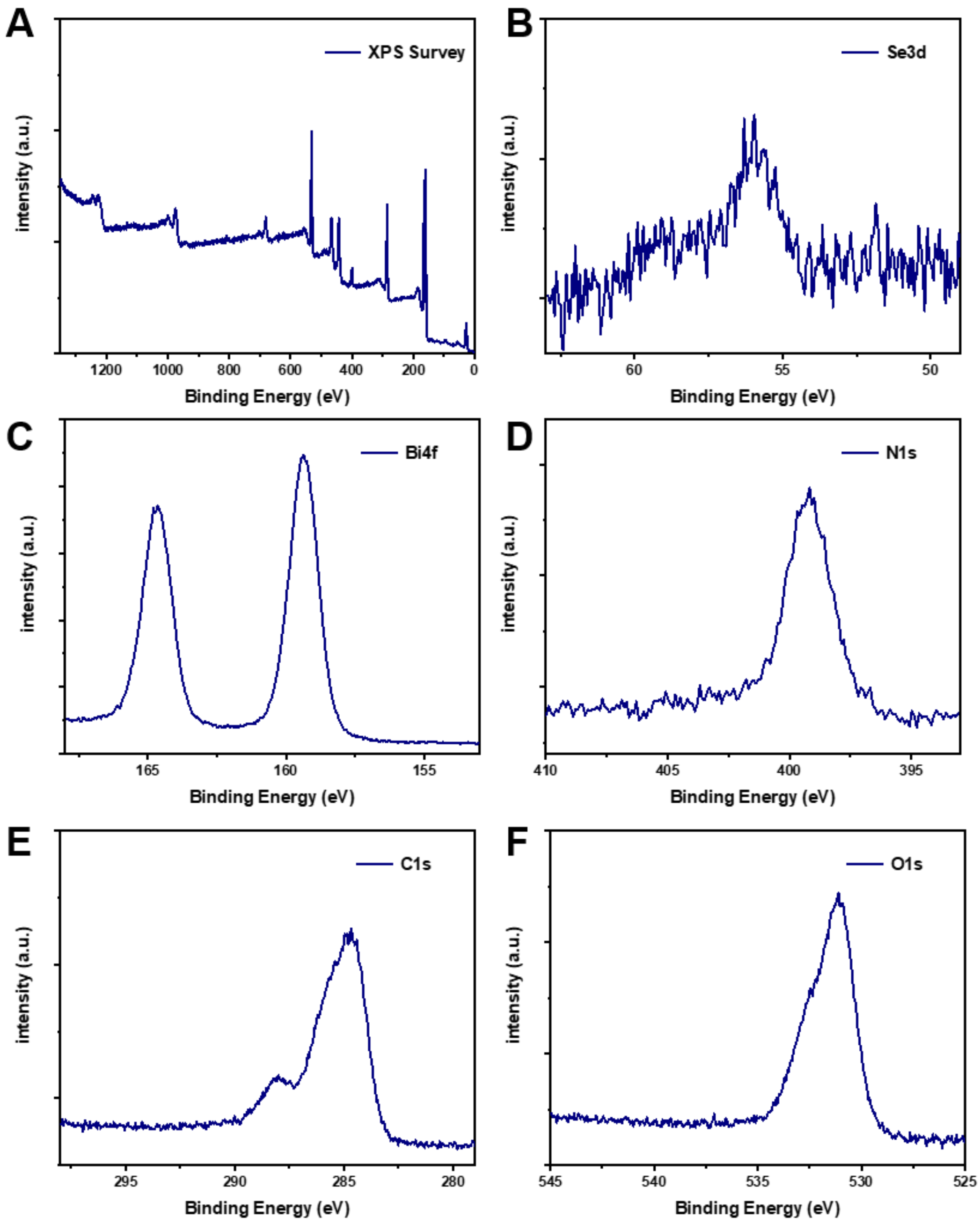

Fig. S1. XPS spectra of MSMN-1. (A) XPS wide-scan survey spectrum. **(B)** Se 3d spectrum. **(C)** Bi 4f spectrum. **(D)** N 1s spectrum. **(E)** C 1s spectrum. **(F)** O 1s spectrum.

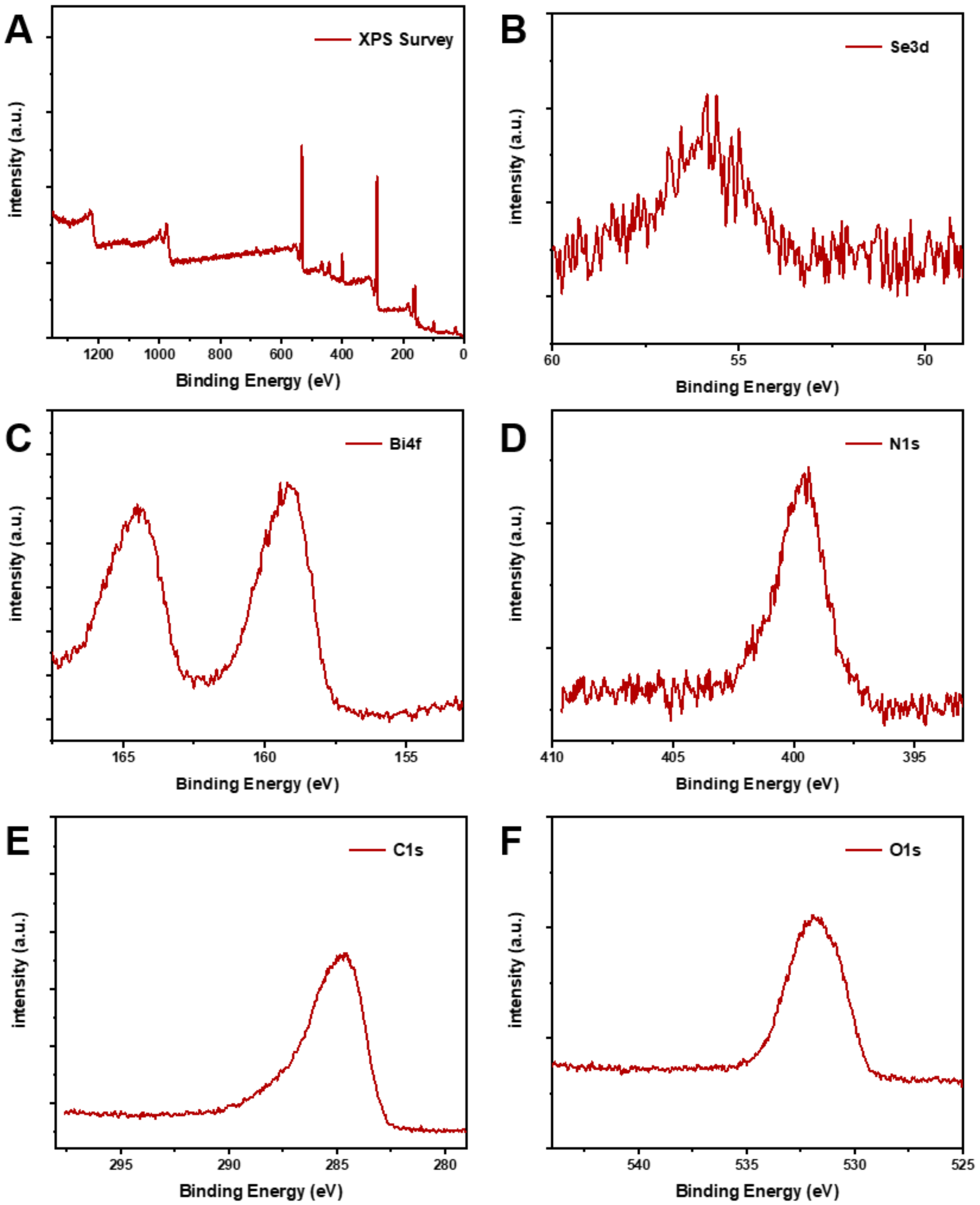

Fig. S2. XPS spectra of MSMN-2. (A) XPS wide-scan survey spectrum. **(B)** Se 3d spectrum. **(C)** Bi 4f spectrum. **(D)** N 1s spectrum. **(E)** C 1s spectrum. **(F)** O 1s spectrum.

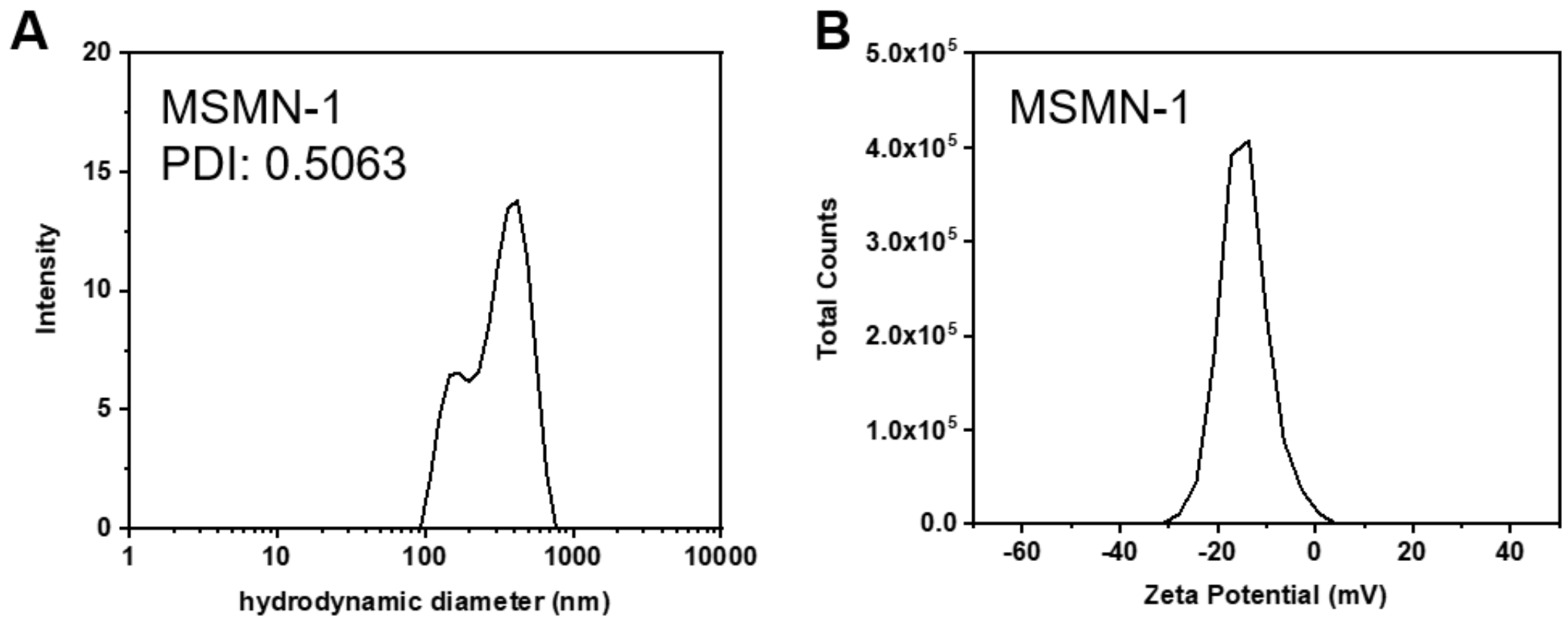

Fig. S3. DLS intensity plots of MSMN-1. (A) Hydrodynamic diameter. **(B)** Zeta potential.

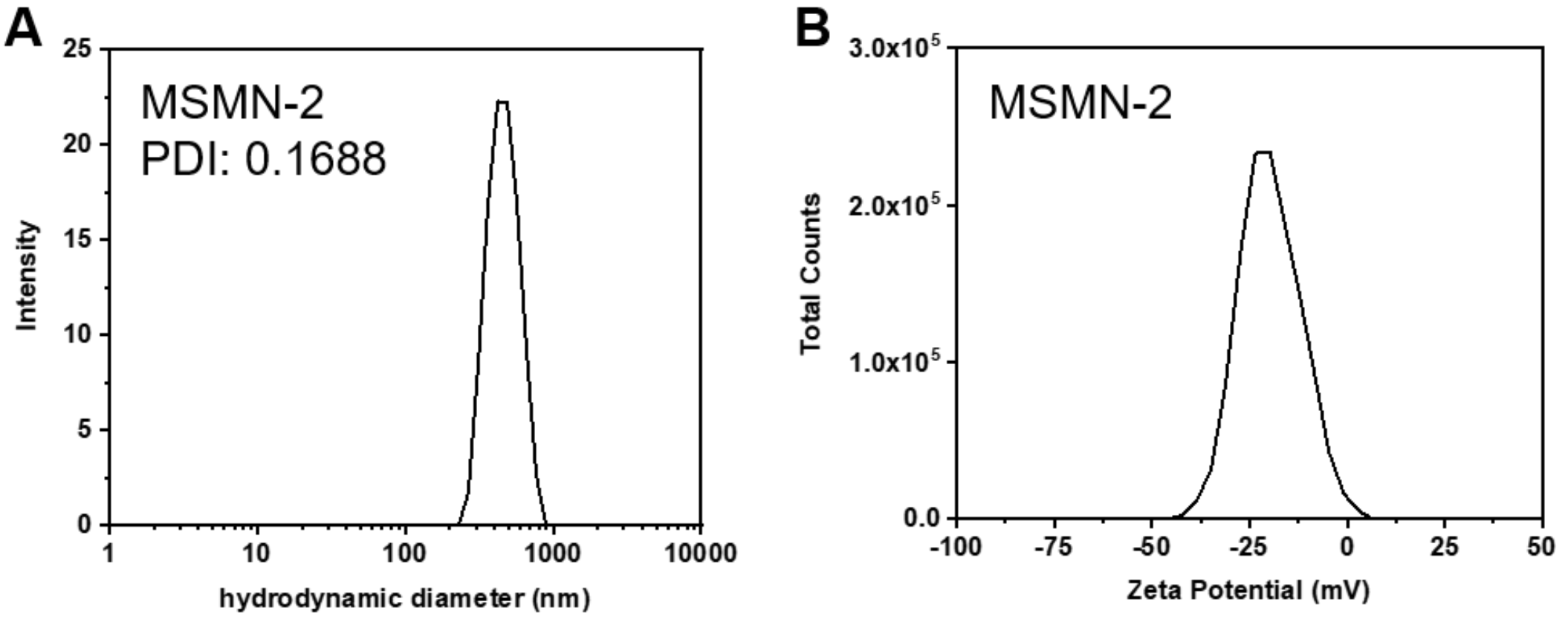

Fig. S4. DLS intensity plots of MSMN-2. (A) Hydrodynamic diameter. **(B)** Zeta potential.

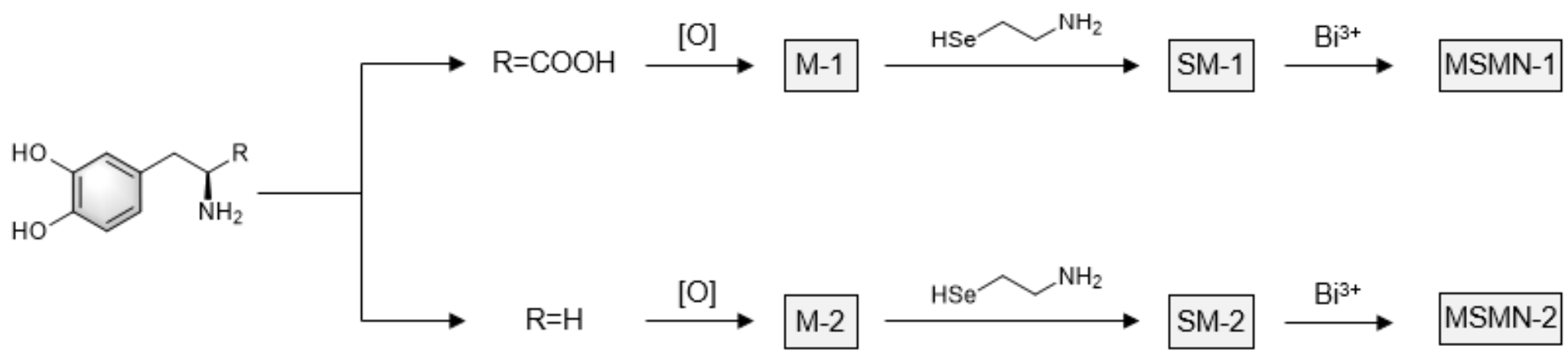

Fig. S5. The synthetic routes of the control melanins and the experimental melanin used in this study.

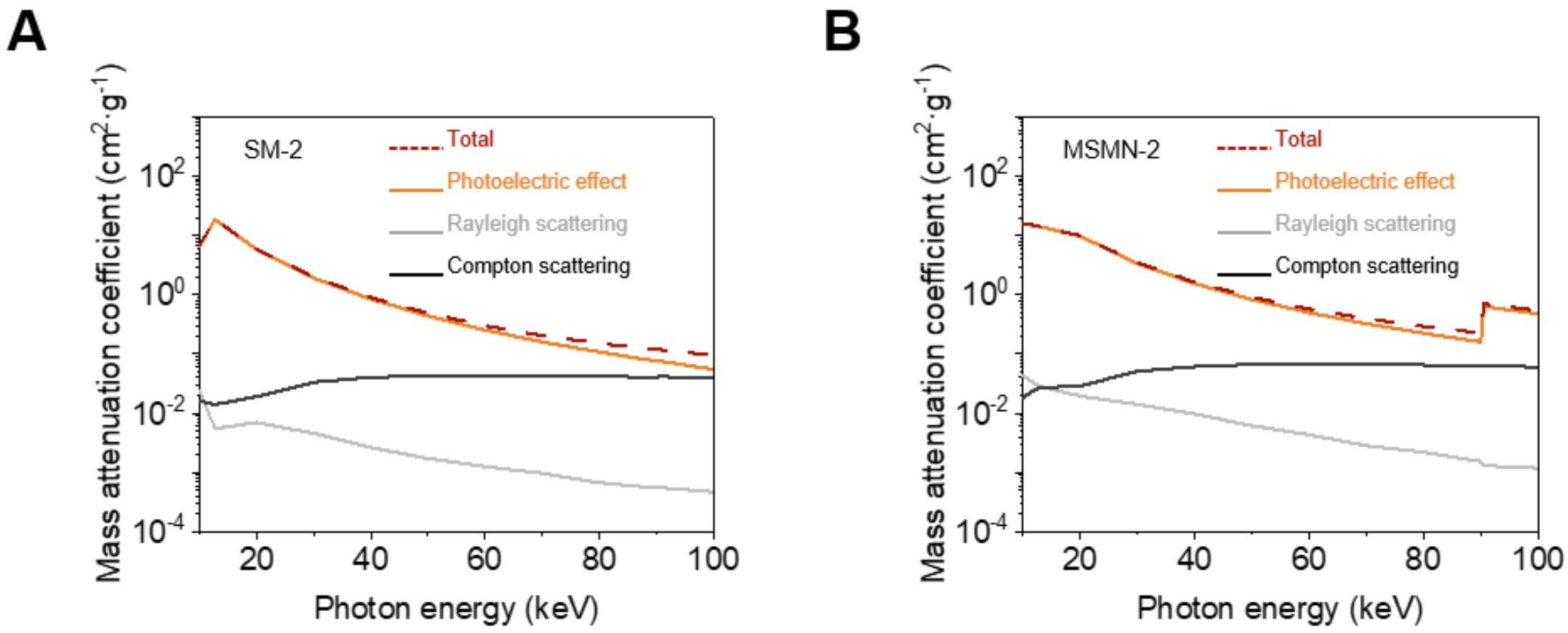

Fig. S6. Component mass attenuation curves obtained by Geant4-based Monte Carlo simulation. (A) Component mass attenuation curves of SM-2. **(B)** Component mass attenuation curves of MSMN-2. Note that both x- and y-axis are logarithmic scales.

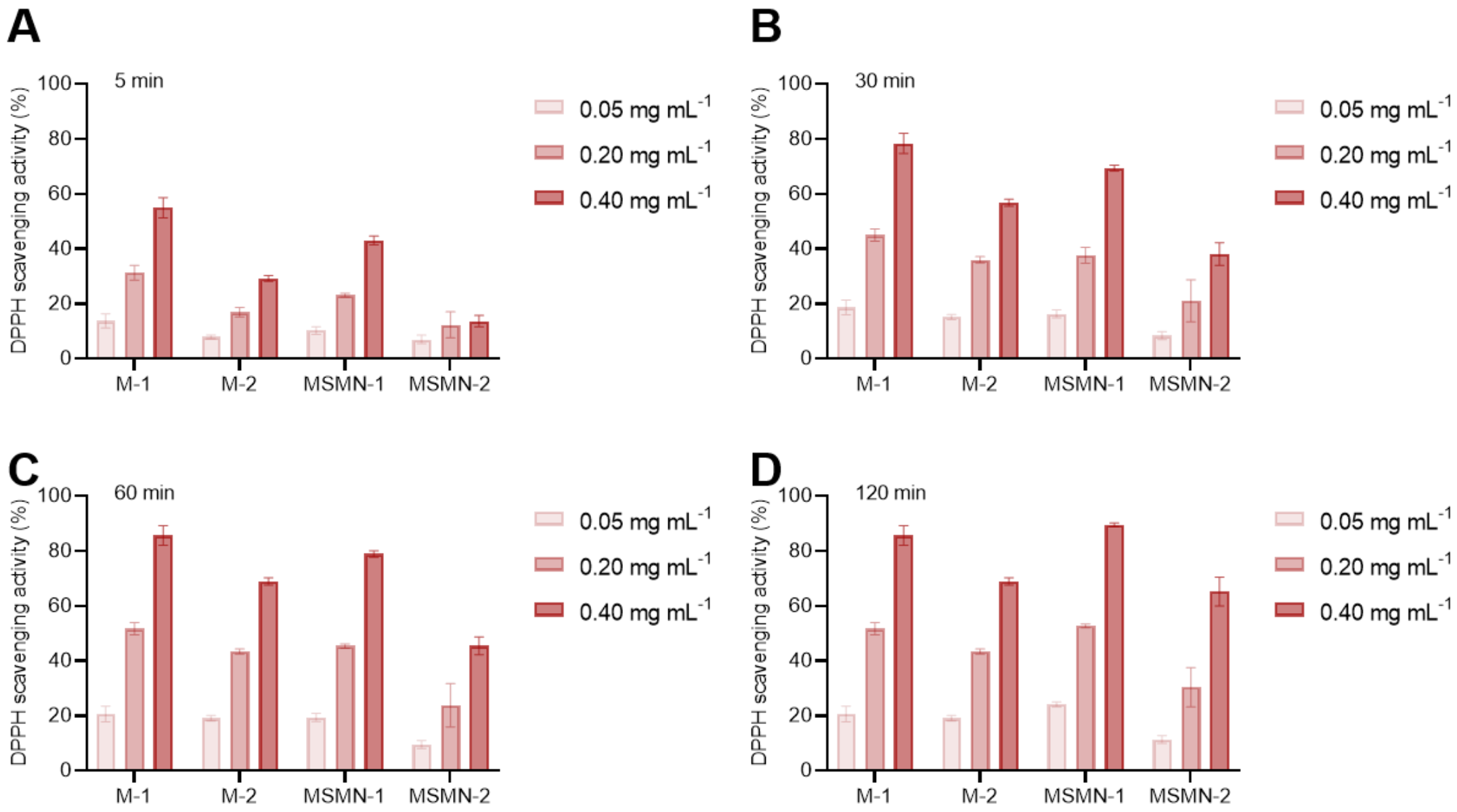

Fig. S7. DPPH scavenging activity of various melanin at different concentrations over time. (A) 5 min. **(B)** 30 min. **(C)** 60 min. **(D)** 120 min.

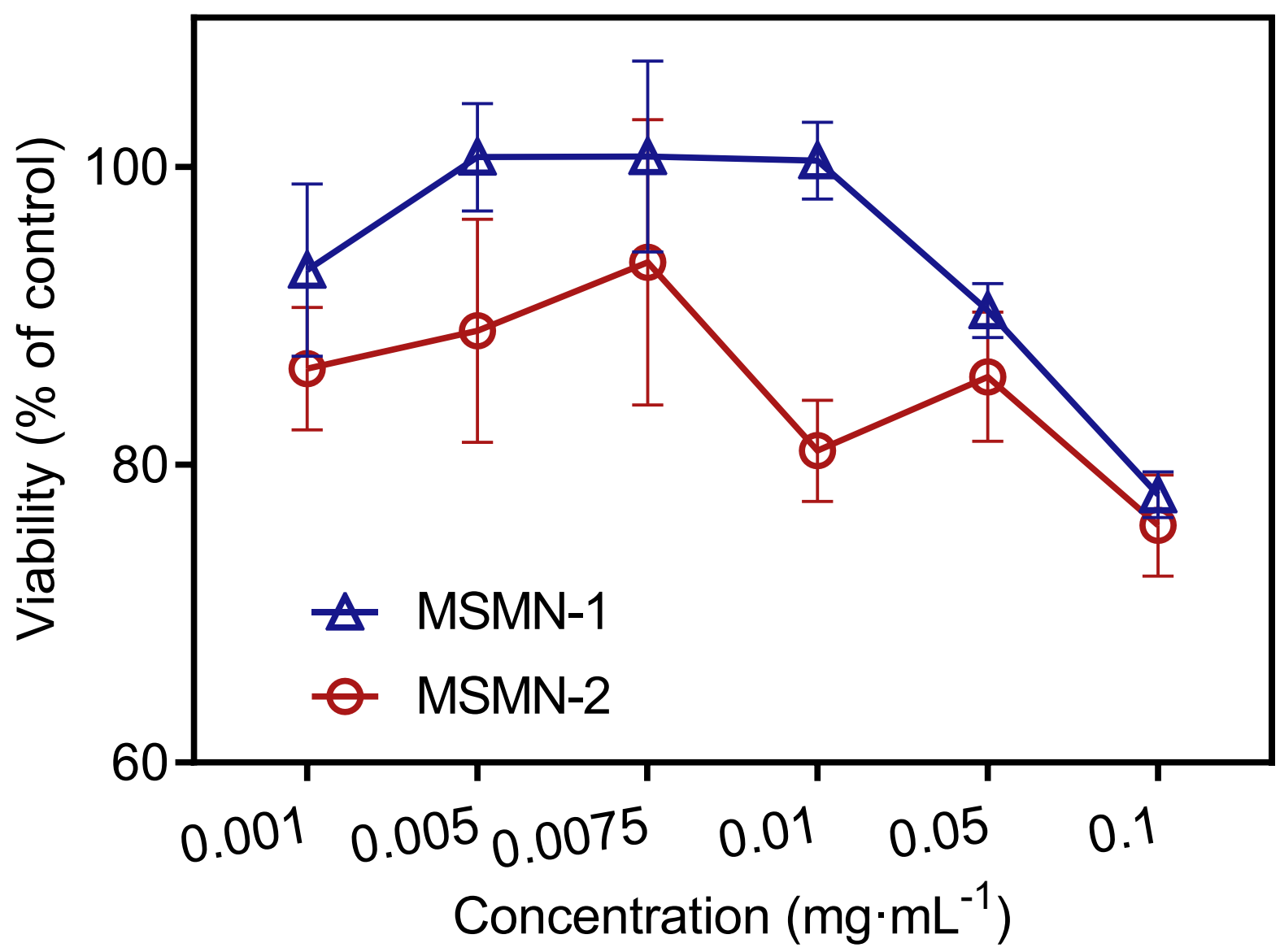

Fig. S8. Cell viability of HaCaT cells after incubation with different concentrations of MSMN for 24 h (*n*=5).

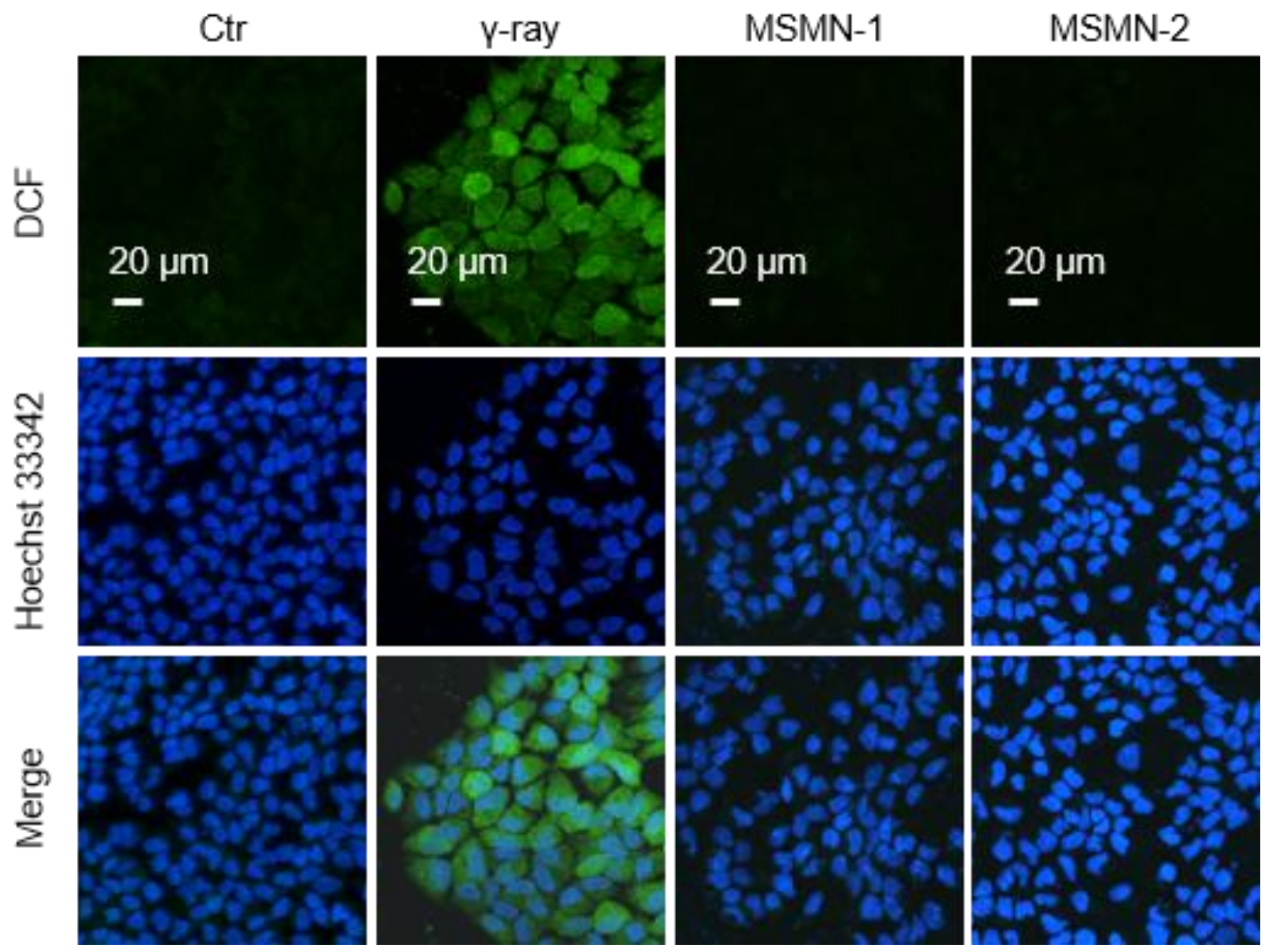

Fig. S9. CLSM images of live cells treated with 10 Gy γ-ray, showing DCF-DA ROS-positive signal (green). Cell nuclei were stained with Hoechst 33342 (blue). Scale bar: 20 μm. ROS probe scan excitation wavelength: 488 nm. Detection wavelength: 510-550 nm. Hoechst 33342 scan excitation wavelength: 405 nm. Detection wavelength: 420-480 nm. Note: Single-channel data from this imaging field is also shown in Fig. 4C.

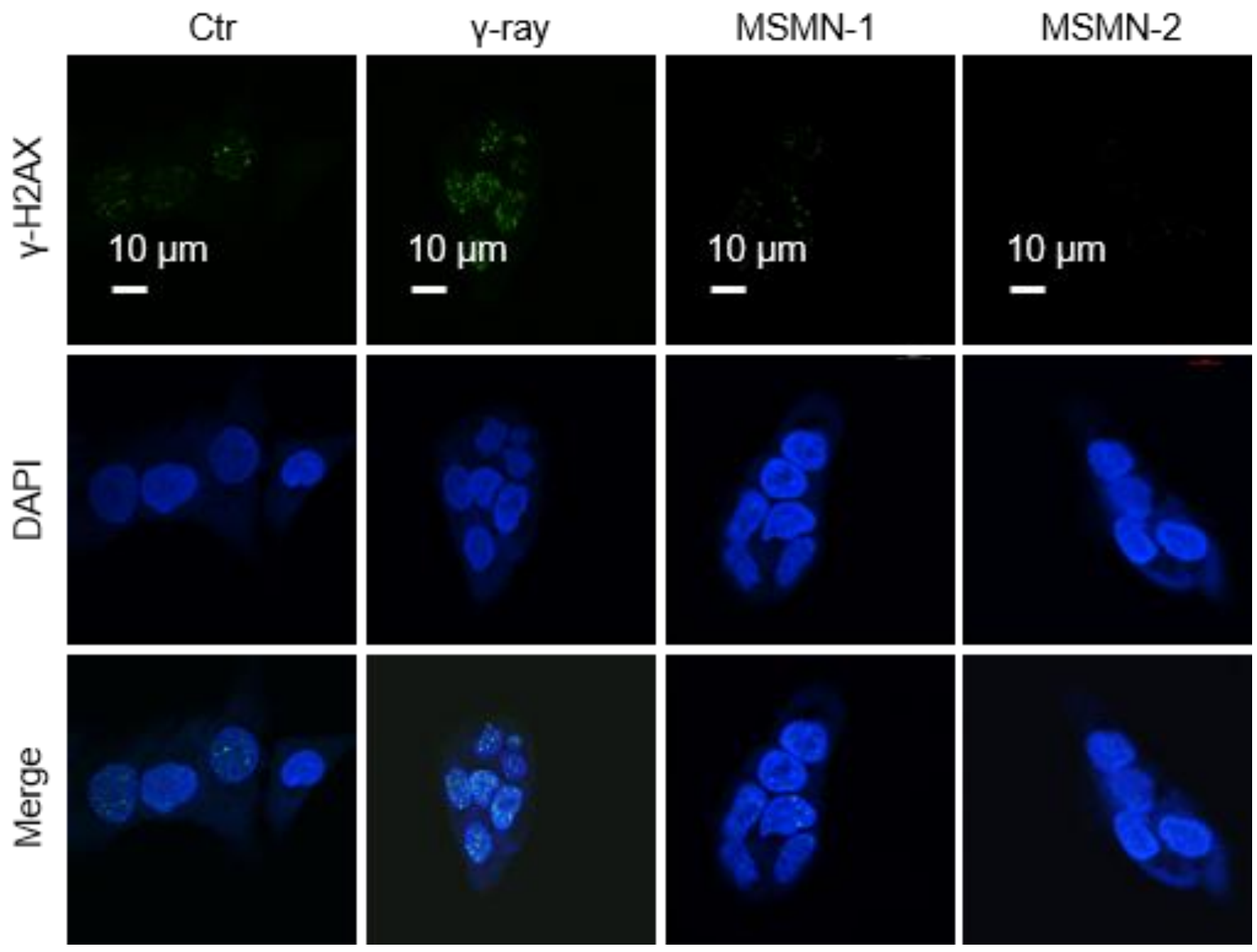

Fig. S10. DNA damage (γ-H2AX foci, green) of HaCaT cells treated with 10 Gy γ-ray. Cell nuclei were stained with DAPI (blue). Scale bar: 10 μm. γ-H2AX scan excitation wavelength: 488 nm. Detection wavelength: 510-550 nm. DAPI scan excitation wavelength: 405 nm. Detection wavelength: 420-480 nm. Note: Single-channel data from this imaging field is also shown in Fig. 4D.

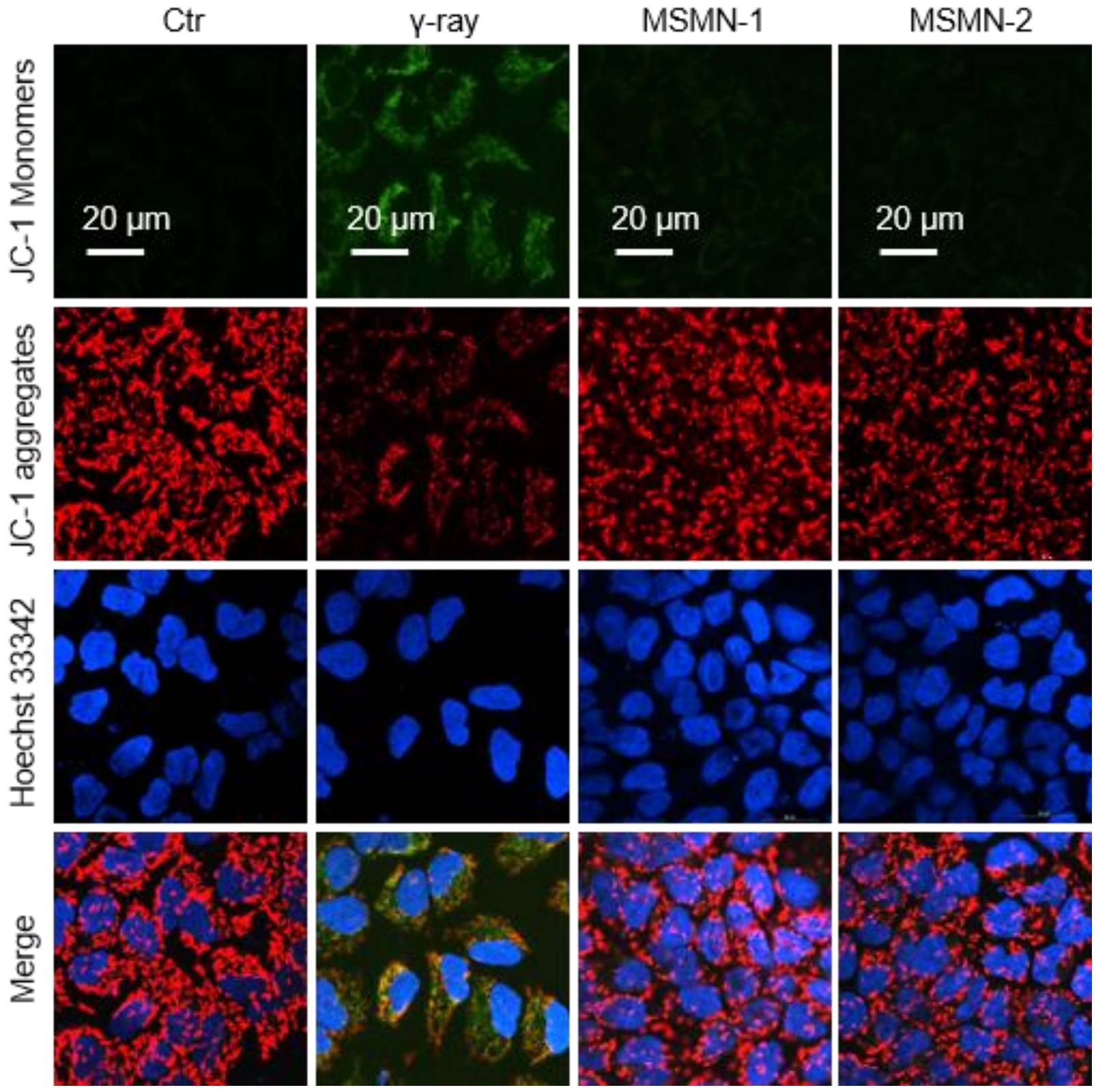

Fig. S11. CLSM images of mitochondrial membranes. JC-1 in high-potential healthy mitochondrial membranes fluoresces red at 561 nm excitation (corresponding to JC-1 aggregates), whereas JC-1 exists as monomers in low-potential mitochondrial membranes and fluoresce green at 488 nm excitation. Cell nuclei were stained with Hoechst 33342 (blue). Scale bar: 20 μm. JC-1 aggregates scan excitation wavelength: 561 nm. Detection wavelength: 570-625 nm. JC-1 monomers scan excitation wavelength: 488 nm. Detection wavelength: 510-550 nm. Hoechst 33342 scan excitation wavelength: 405 nm. Detection wavelength: 420-480 nm. Note: Single-channel data from this imaging field is also shown in Fig. 4E.

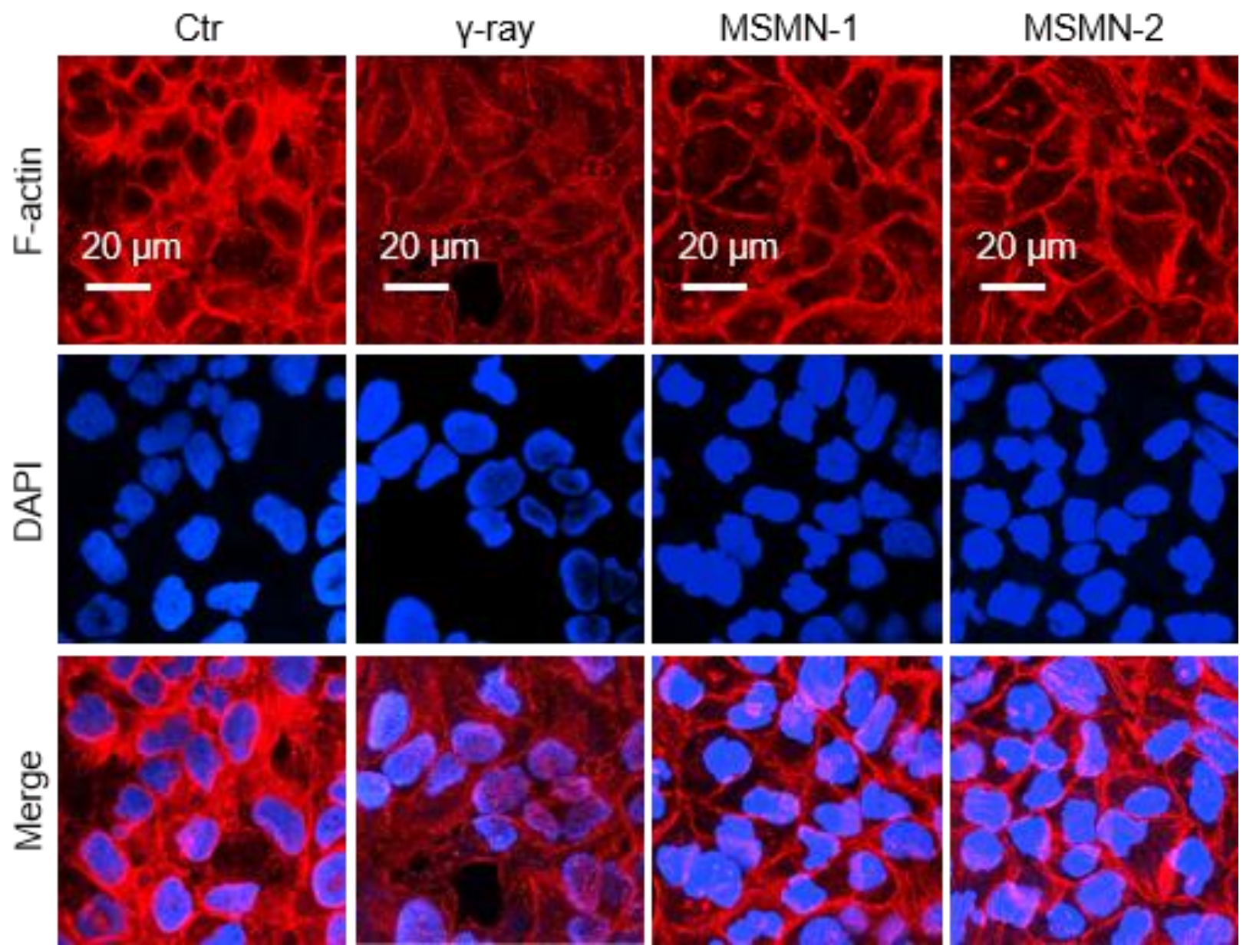

Fig. S12. CLSM images of live cells treated with 10 Gy γ-ray, showing Actin-Tracker Red-594 F-actin-positive signal (red). Cell nuclei were stained with DAPI (blue). Scale bar: 20 μm. F-actin probe scan excitation wavelength: 561 nm. Detection wavelength: 570-625 nm. DAPI scan excitation wavelength: 405 nm. Detection wavelength: 420-480 nm. Note: Single-channel data from this imaging field is also shown in Fig. 4F.

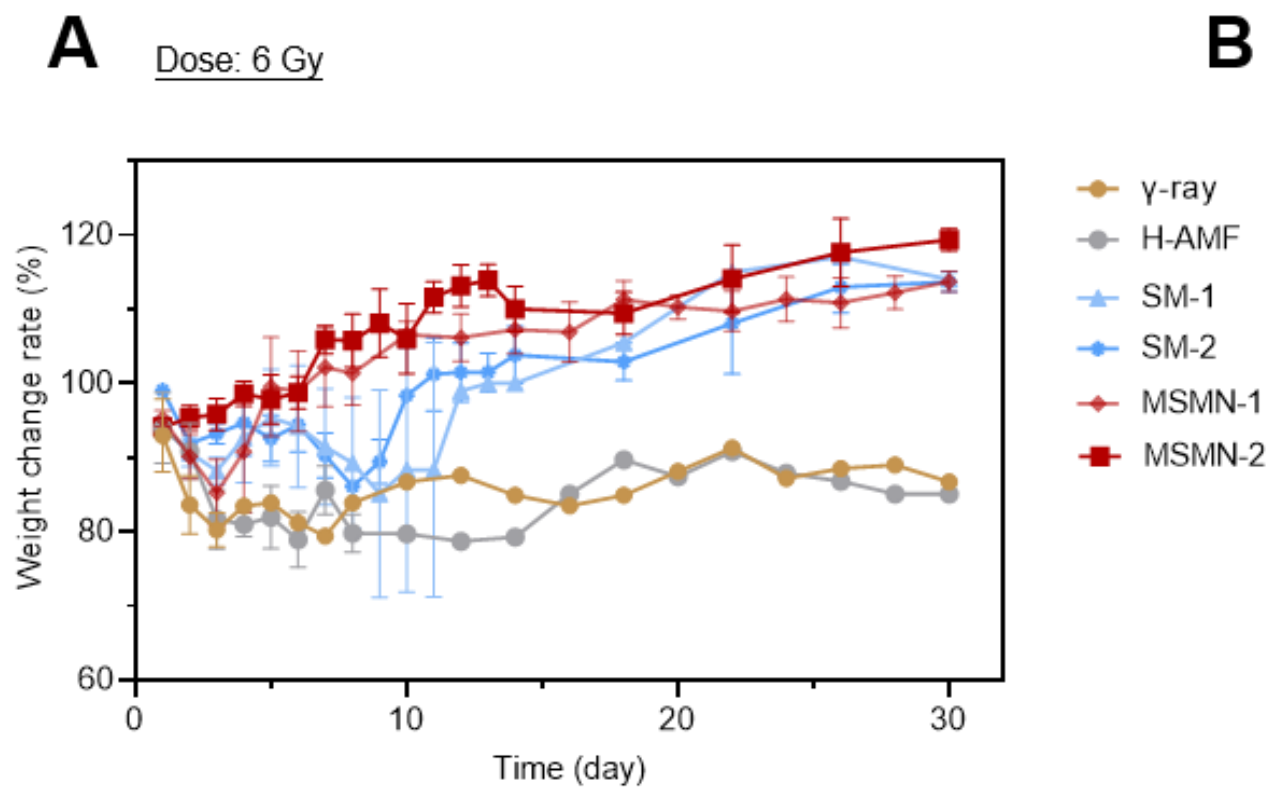

B

Group	Live (%)
γ-ray	12.5
H-AMF	20
SM-1	0
SM-2	100
MSMN-1	100
MSMN-2	100

Fig. S13. 6 Gy total body irradiation model. (A) The body weight changes during different treatments. **(B)** The survival rates of each group on day 30.

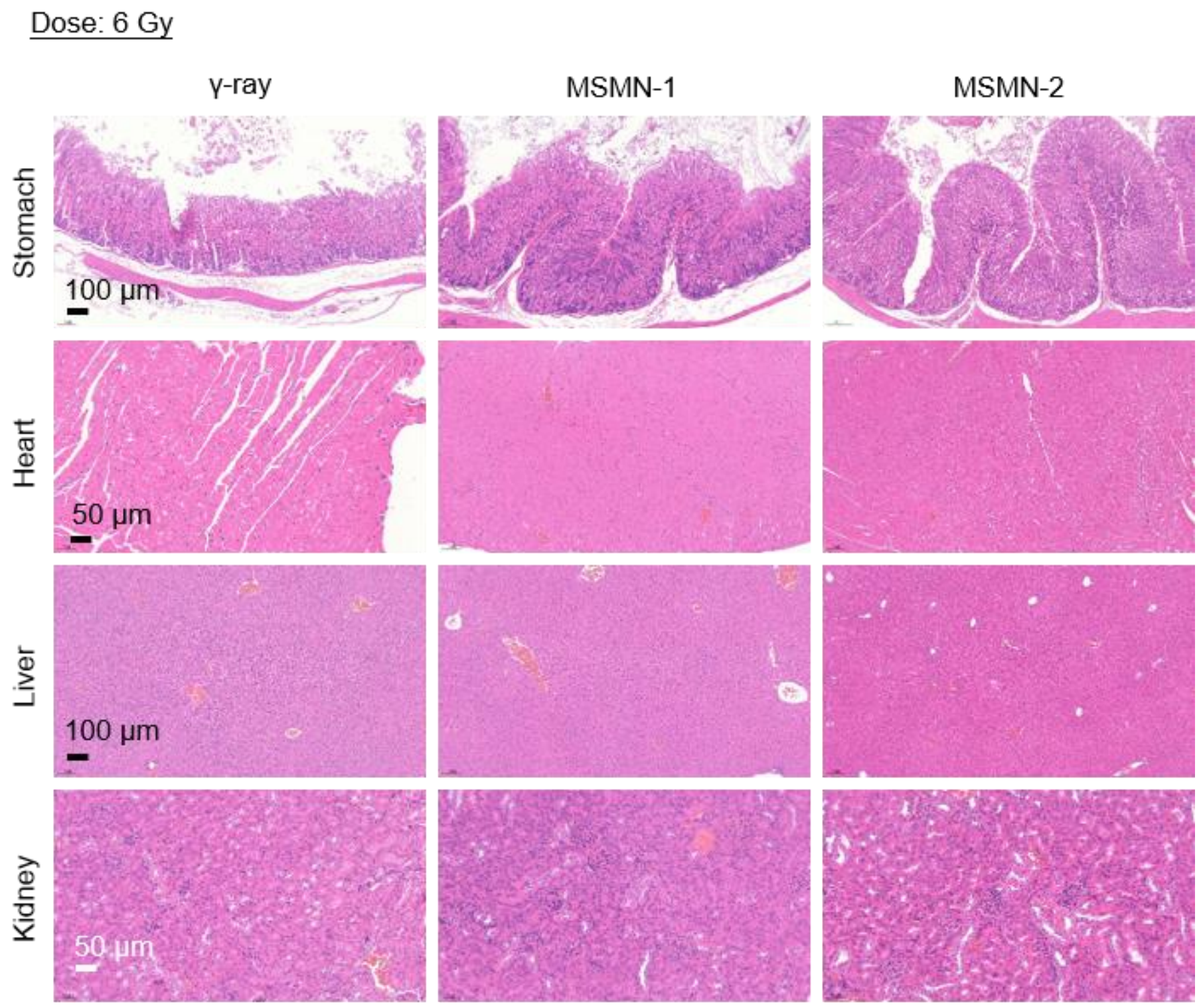

Fig. S14. H&E staining of stomach, kidney, heart and liver at the 30-day censoring point.

Scale bars of heart and kidney: 50 μm. Scale bars of stomach and liver: 100 μm.

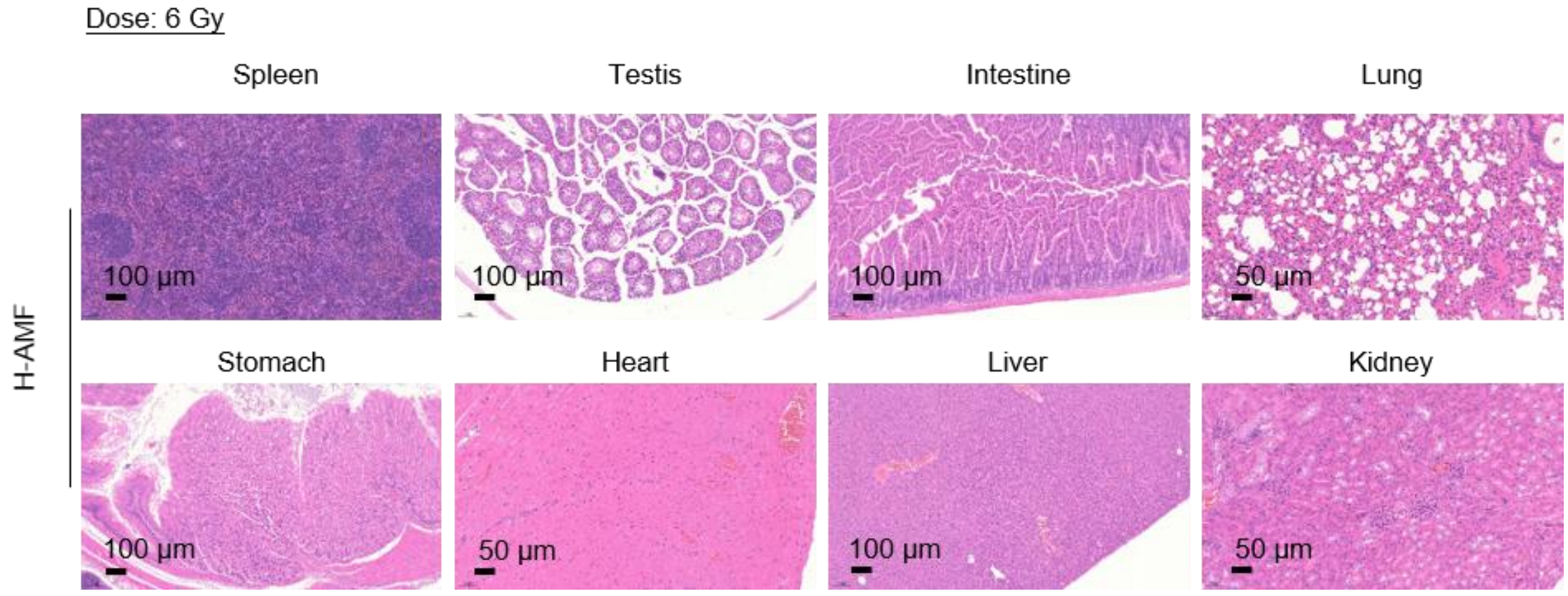

Fig. S15. H&E staining of spleen, testis, lung, intestine, stomach, kidney, heart and liver at the 30-day censoring point. Scale bar of lung, heart and kidney: 50 μm. Scale bar of other organs: 100 μm.

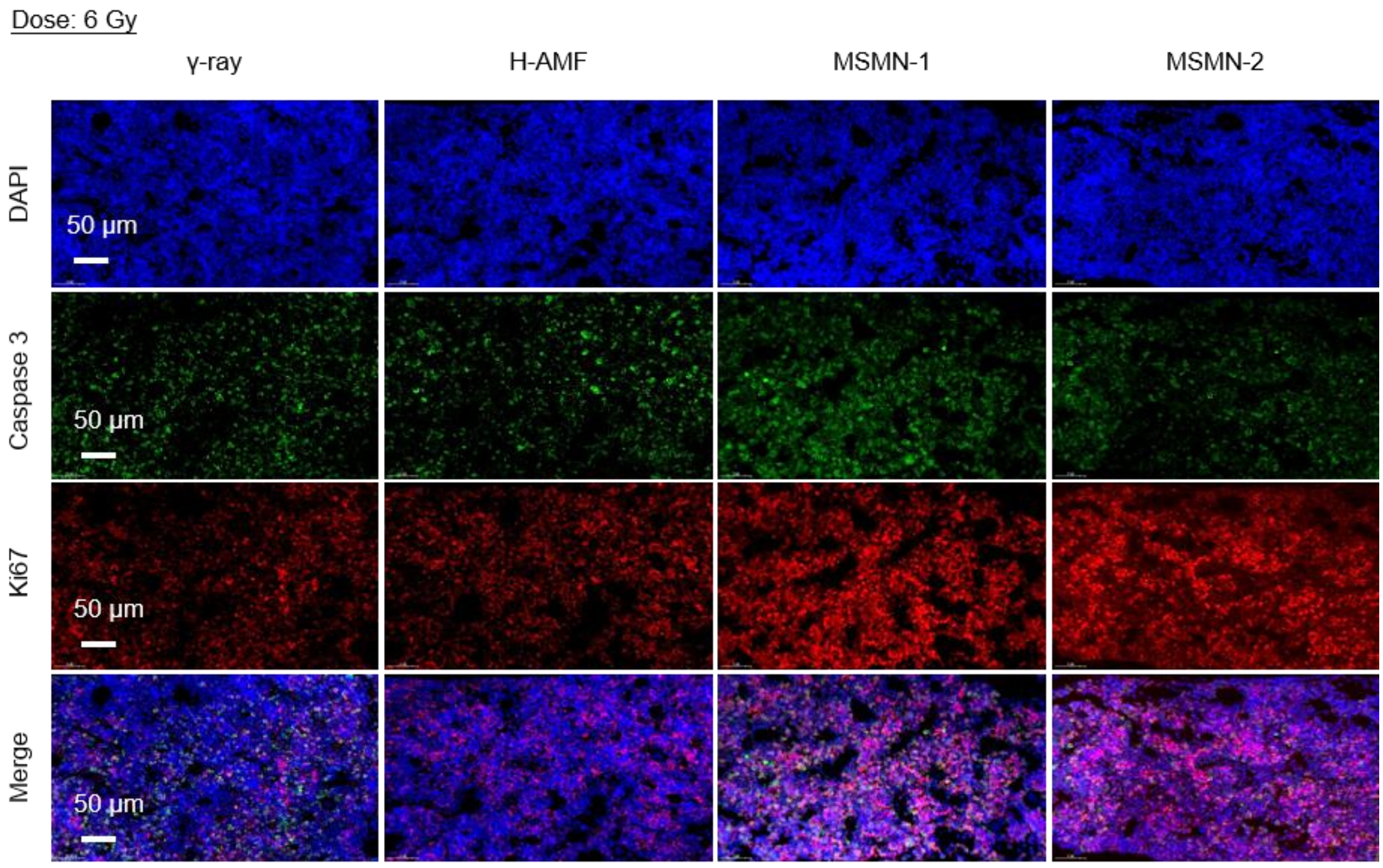

Fig. S16. IF staining of bone marrow at the 30-day censoring point in 6 Gy total body irradiation model. Cell nuclei were stained with DAPI. Hematopoietic cell death was marked by cleaved caspase-3 and proliferation was indicated by Ki67. Scale bar: 50 µm. Note: Single-channel data from this imaging field is also shown in Fig. 5E.

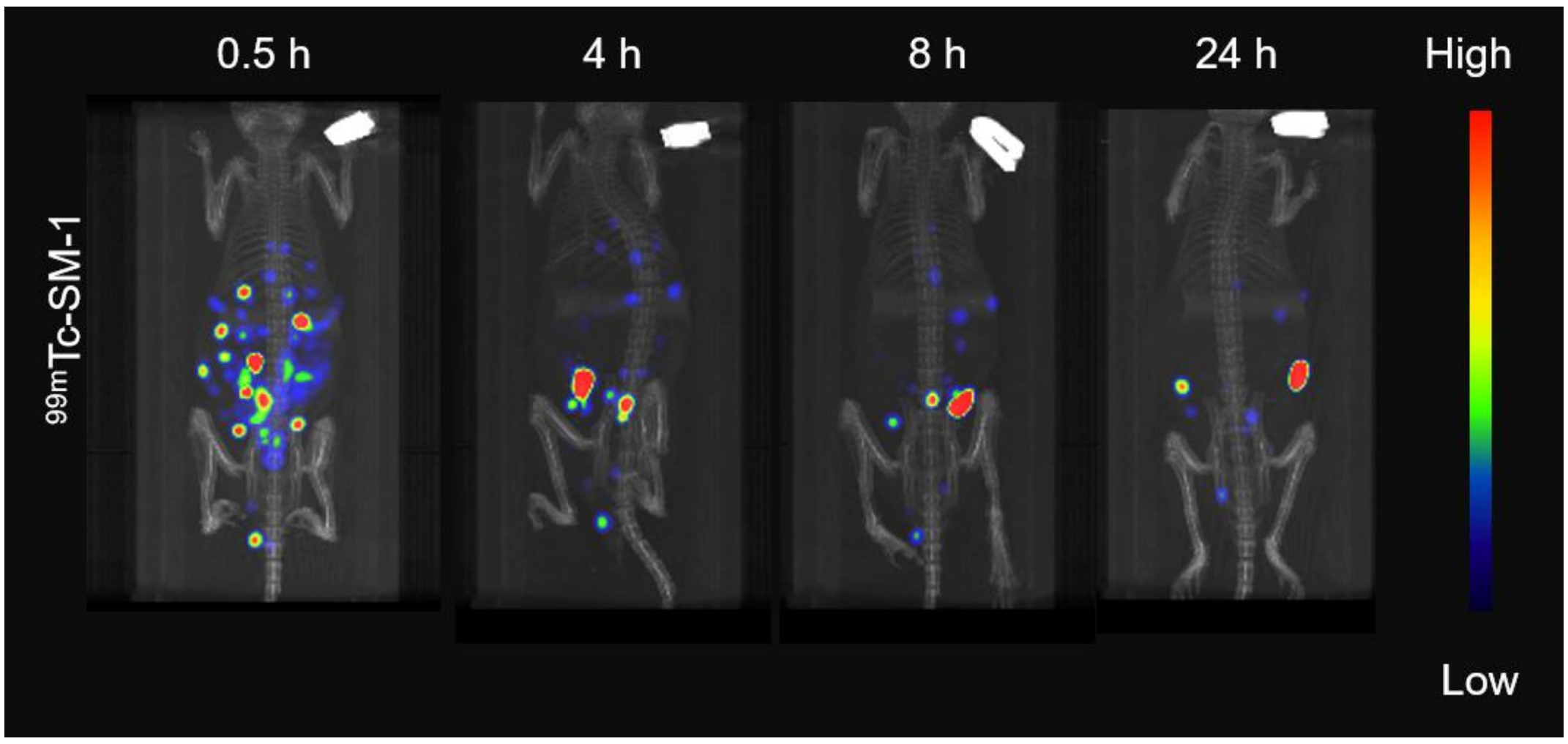

Fig. S17. Whole-body SPECT-CT images of ^{99m}Tc-SM-1 at various time points (0.5 h, 4 h, 8 h and 24 h) post 0.4 mCi ^{99m}Tc-SM-1 administration. The high CT value white area in the image corresponds to the mouse ear tag and should not be mistaken for the thyroid.

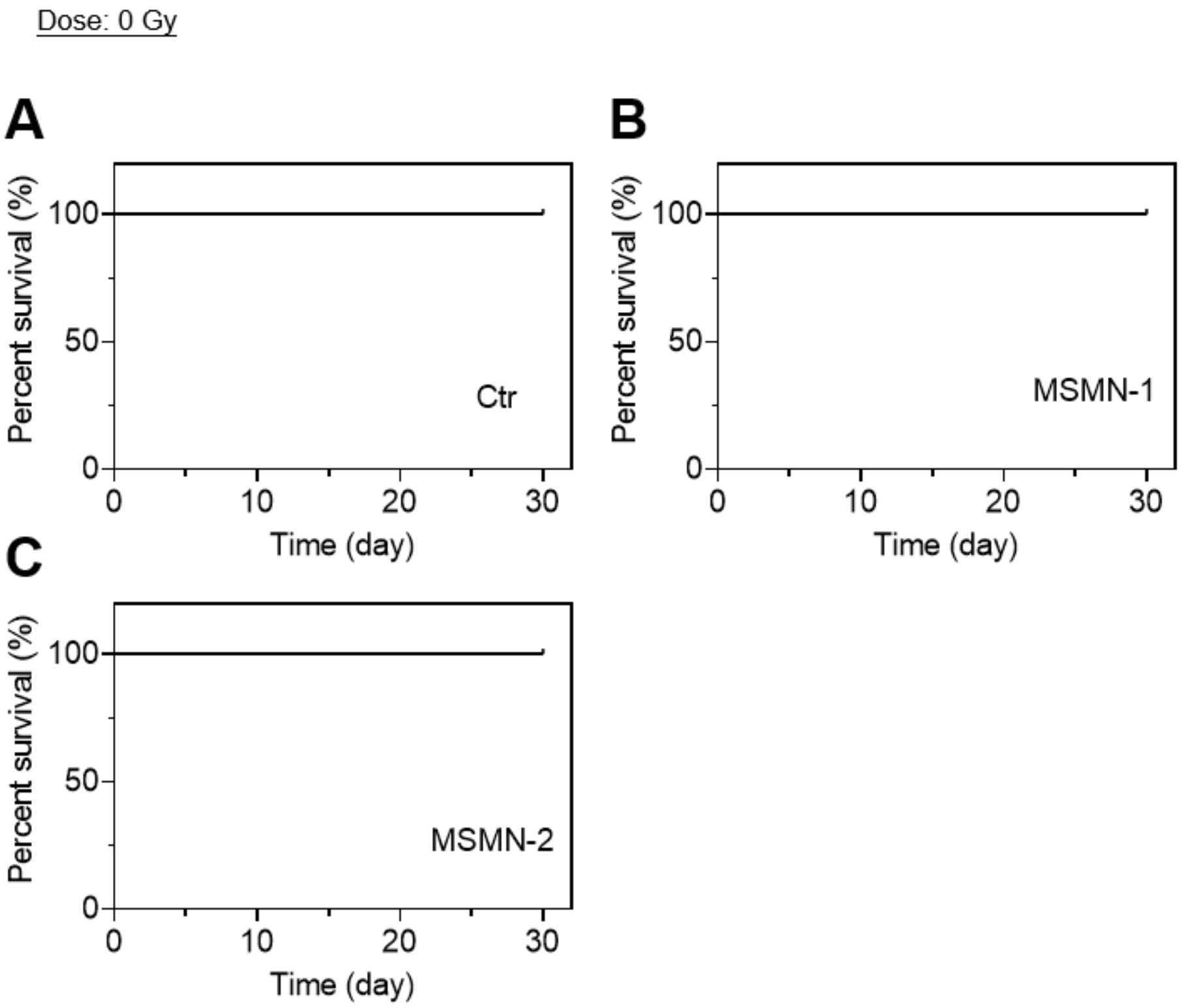

Fig. S18. Monitoring of mice survival 30 days after injection in biosafety testing. The mice were divided into 3 groups: **(A)** Ctr (sham control, *n*=3). **(B)** MSMN-1 (*n*=3). **(C)** MSMN-2 (*n*=3). Groups **(B-C)** each received an intraperitoneal injection of 7.5 $mg \cdot kg^{-1}$ the respective test agent.

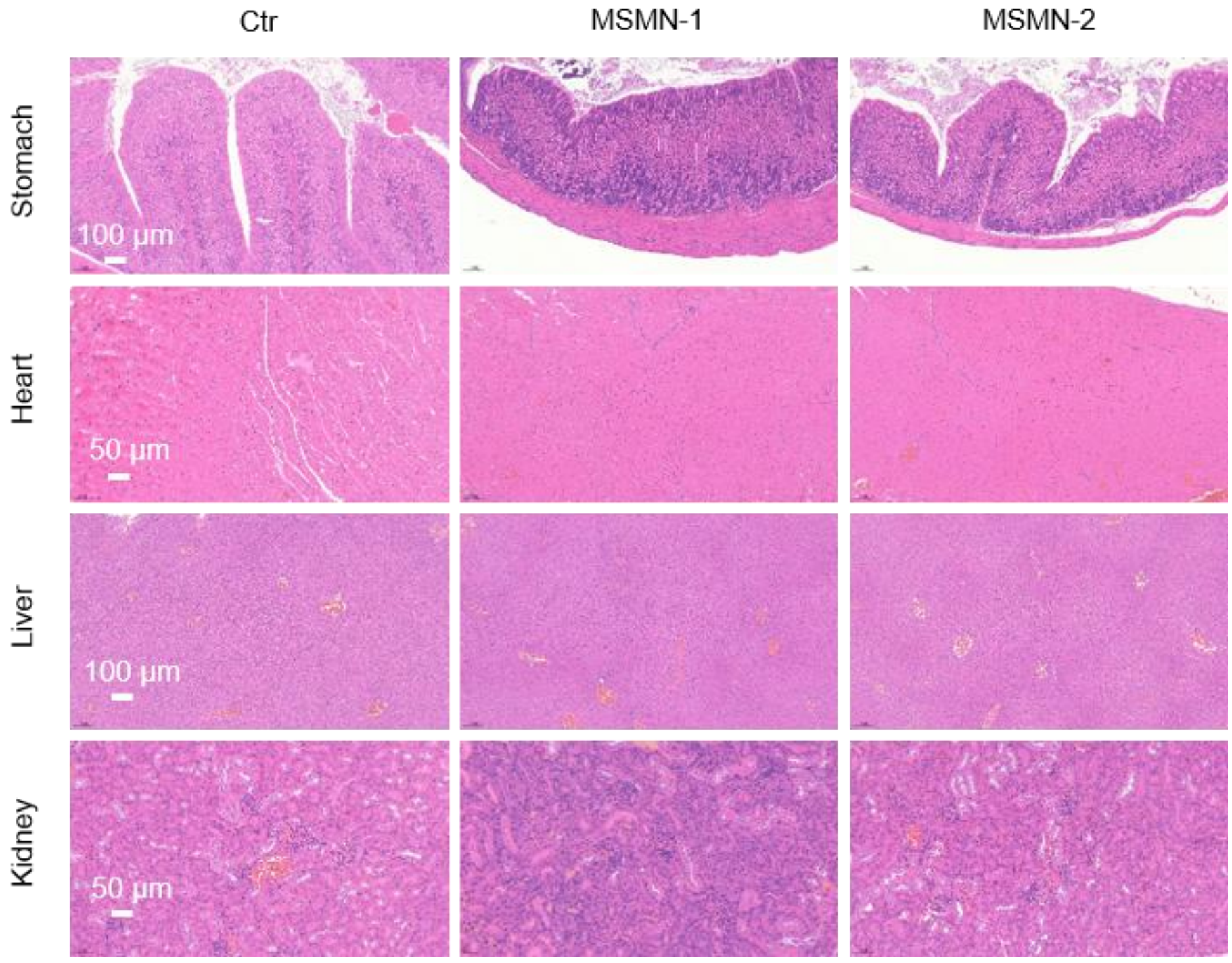

Fig. S19. H&E staining of stomach, kidney, heart and liver at the 30-day censoring point in biosafety testing. Scale bar of heart and kidney: 50 μm. Scale bar of stomach and liver: 100 μm.

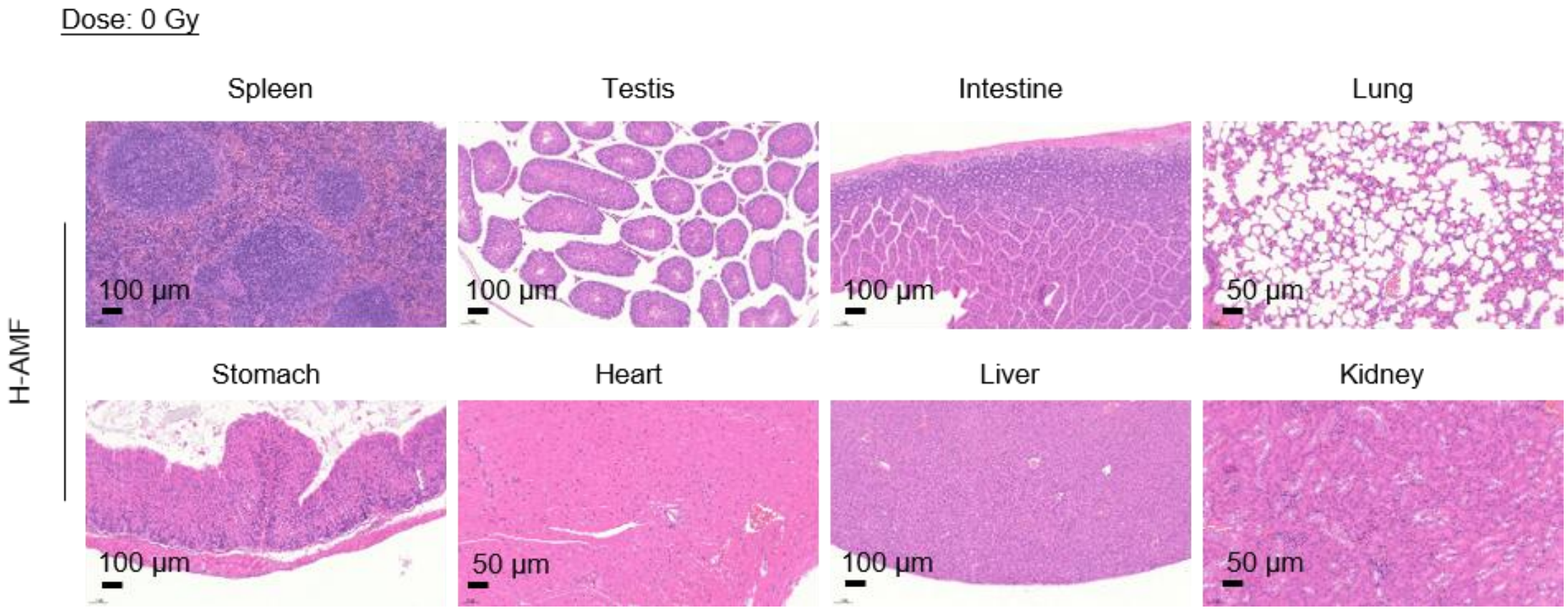

Fig. S20. H&E staining of spleen, testis, lung, intestine, stomach, kidney, heart and liver at the 30-day censoring point in biosafety testing. Scale bar of lung, heart and kidney: 50 µm. Scale bar of other organs: 100 µm.

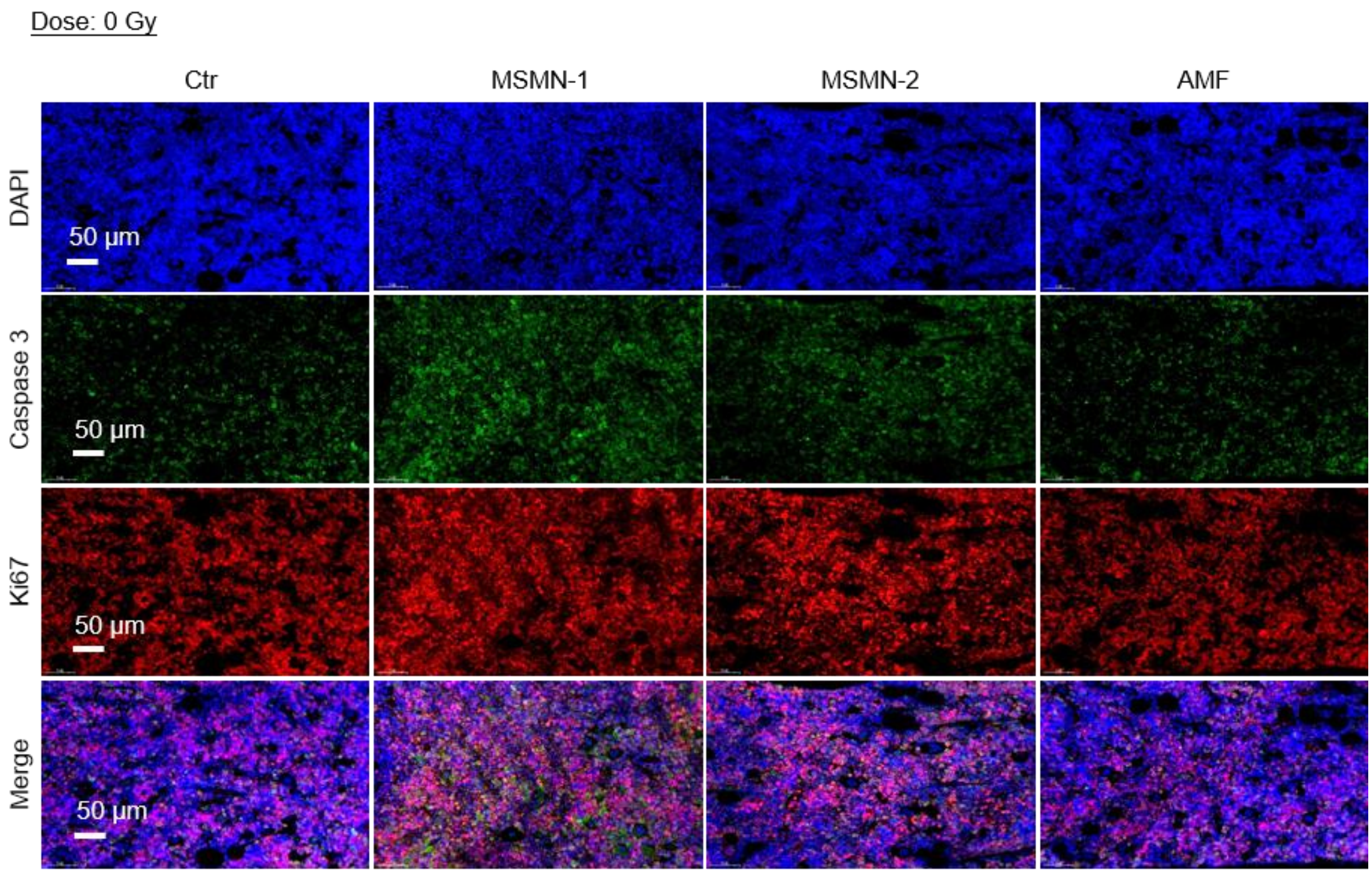

Fig. S21. IF staining of bone marrow at the 30-day censoring point in biosafety testing. Cell nuclei were stained with DAPI. Hematopoietic cell death was marked by caspase-3 and proliferation was indicated by Ki67. Scale bar: 50 μm. Note: Single-channel data from this imaging field is also shown in Fig. 6E.

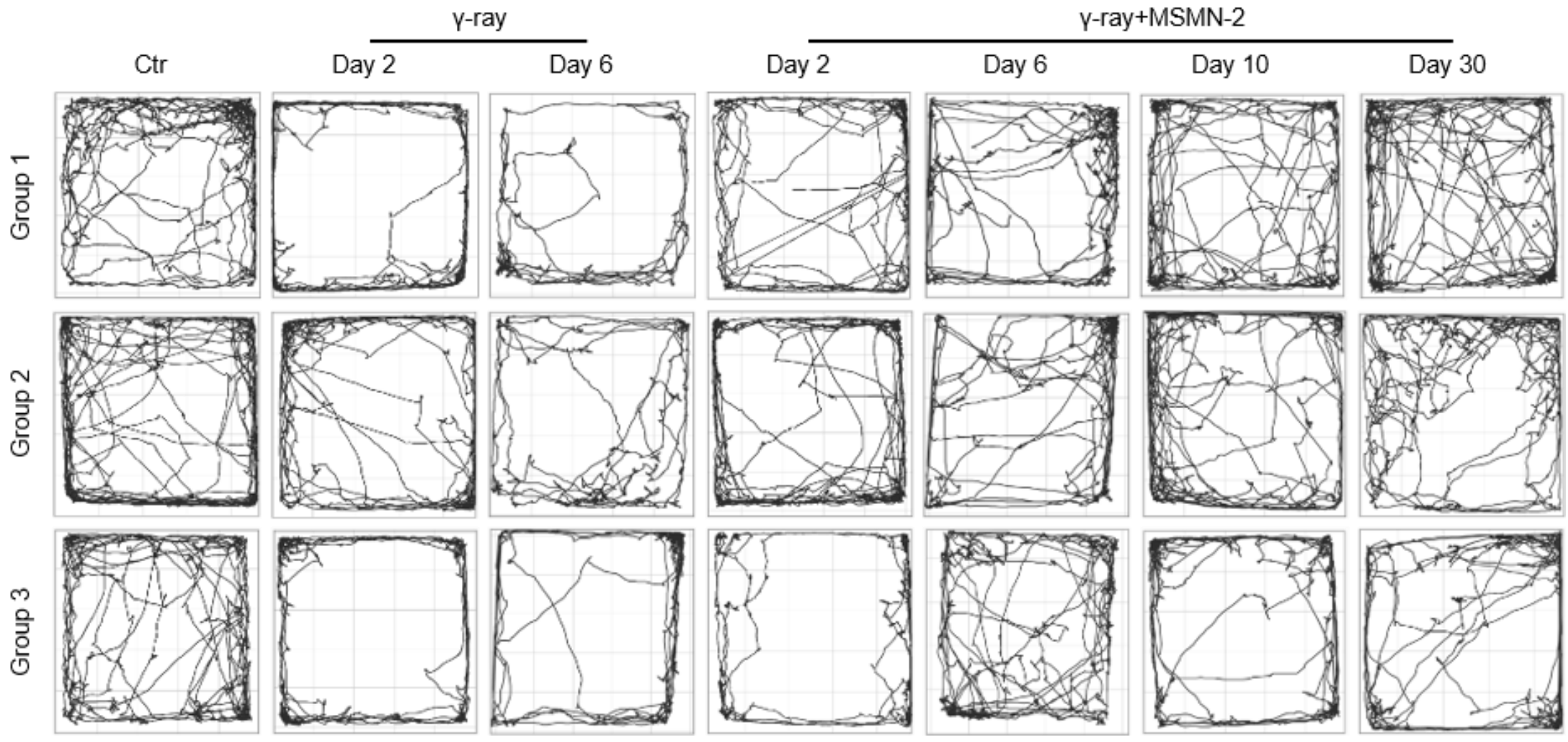

Fig. S22. Movement trajectories in the open field test. Groups 1–3 denote data from three biological replicates (*n* = 3). The mice were divided into 3 groups: (I) Ctr (sham control, *n*=3). (II)γ-ray (only 6 Gy total body irradiation, *n*=3). (III) γ-ray+MSMN-2 (*n*=3). All irradiated groups [Groups (II) and (III)] received total body irradiation (6 Gy, 2 Gy·min^{-1}, 3 min). Group (III) was intraperitoneally injected with 3.75 mg·kg^{-1} MSMN-2 2 h before and 24 h after the 6 Gy total body irradiation (cumulative dose 7.5 mg·kg^{-1}). In Group (II), the open field test was conducted on the 2nd and 6th days after irradiation. In Group (III), the open field test was conducted on the 2nd, 6th, 10th and 30th days after irradiation. Mice used in the open field test were not from the same cohort as those for the above survival assay and subsequent striatal discharge recordings. In this cohort, none of the irradiated mice (*n*=3) survived to 30 days; therefore, no data were available for the γ-ray-30-day group.

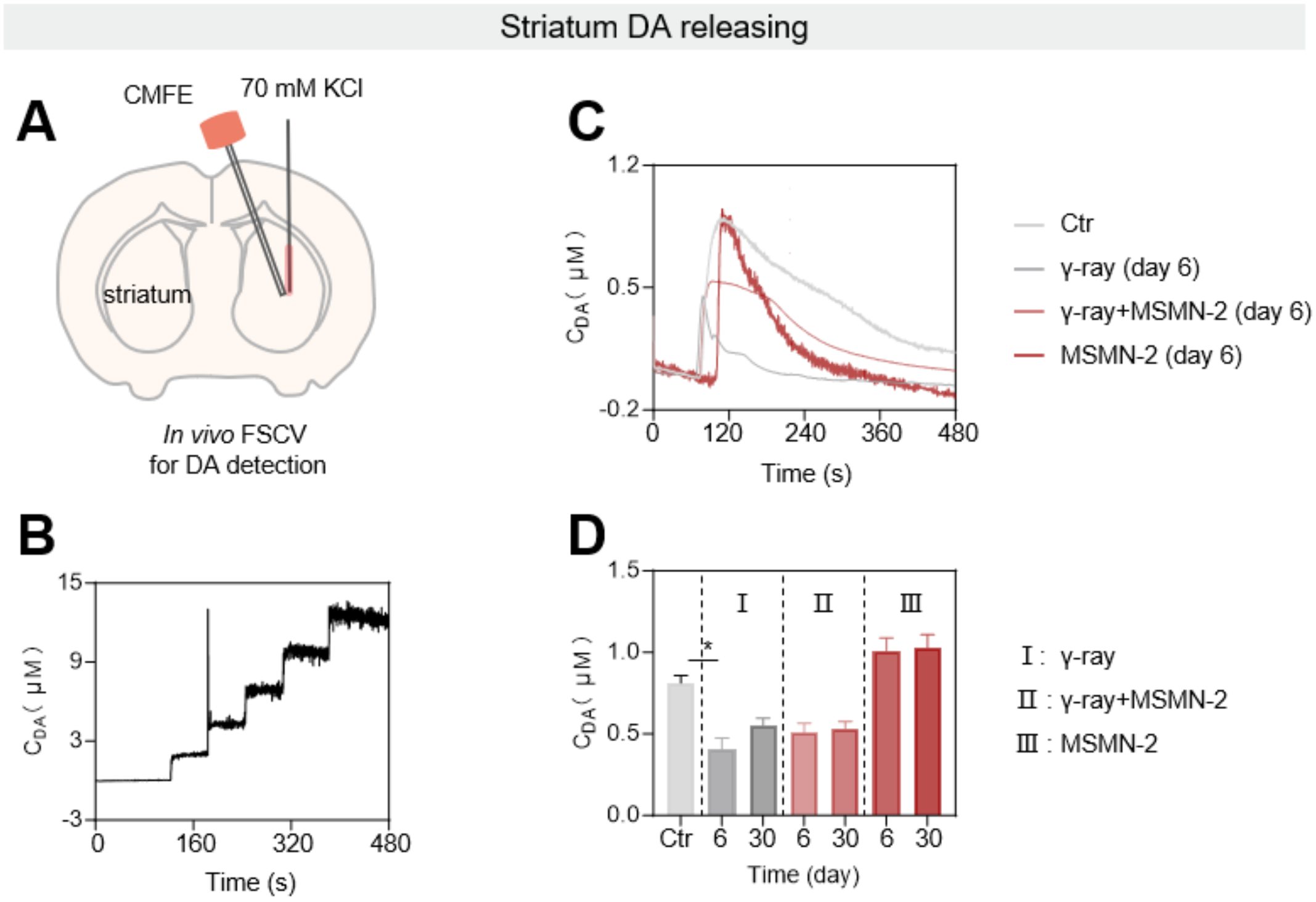

Fig. S23. Quantification of KCl-evoked striatal dopamine (DA) release *via in vivo* fast-scan cyclic voltammetry (FSCV). (A) Schematic illustration of the *in vivo* FSCV setup for detecting 70 mM KCl-stimulated striatal DA release in mouse striatum. A drug delivery cannula was implanted 300 µm away from the carbon fiber electrode at the same depth for local KCl infusion. Created in BioRender. Deng, R. (2026) https://BioRender.com/sn1ce9m. **(B)** Representative linear calibration curve of carbon fiber electrodes (CFEs) for DA quantification. **(C)** Dynamic DA release traces evoked by local infusion of 70 mM KCl in control, γ-ray irradiation, and γ-ray+MSMN-2 co-treatment groups at day 6 post 6 Gy whole-body irradiation. **(D)** Statistical summary of average KCl-triggered striatal DA release concentration measured at day 6 and day 30 for all experimental groups.

Supplementary Tables

Table S1. Elemental mass percentages of samples. The mass percentages of each element were calculated from the atomic percentages obtained *via* XPS, and these values were employed as the input for the calculation of effective atomic number (Z_{eff}) to evaluate the radiation attenuation performance.

Sample	C (wt%)	N (wt%)	O (wt%)	Se (wt%)	Bi (wt%)
SM-1	47.50	9.73	18.23	24.54	0.00
SM-2	49.00	7.78	24.10	19.15	0.00
MSMN-1	34.22	5.15	21.37	5.67	33.60
MSMN-2	53.74	8.00	25.07	4.50	8.69

Table S2. Bi Content (from ICP-OES) of MSMN.

Sample	Bi ($mg \cdot g^{-1}$)
MSMN-1	292.216
MSMN-2	106.664

Table S3. Full pairwise Log-rank comparison results of Kaplan–Meier survival curves presented in Figure 5B. Pairwise Log-rank tests were conducted for all six experimental groups. Bonferroni correction was applied to account for multiple comparisons, with the corrected significance threshold set at 0.05/15 ≈ 0.0033. ns, not significant (*P* > 0.0033); *, statistically significant (*P* < 0.0033).

Group 1	Group 2	*P*-value	Significance (Bonferroni corrected)
γ-ray	H-AMF	0.038	n.s.
γ-ray	SM-1	<0.001	*
γ-ray	SM-2	<0.001	*
γ-ray	MSMN-1	<0.001	*
γ-ray	MSMN-2	<0.001	*
H-AMF	SM-1	0.007	n.s.
H-AMF	SM-2	<0.001	*
H-AMF	MSMN-1	<0.001	*
H-AMF	MSMN-2	<0.001	*
SM-1	SM-2	<0.001	*
SM-1	MSMN-1	<0.001	*
SM-1	MSMN-2	<0.001	*
SM-2	MSMN-1	1.000	ns
SM-2	MSMN-2	1.000	ns
MSMN-1	MSMN-2	1.000	ns
